# Combined the Photocatalysis and Fenton-like Reaction to Efficiently Remove Sulfadiazine in Water Using g-C_3_N_4_/Ag/γ-FeOOH: Insights Into the Degradation Pathway From Density Functional Theory

**DOI:** 10.3389/fchem.2021.742459

**Published:** 2021-10-05

**Authors:** Yangchen Zhu, Furong Zhao, Fei Wang, Beihai Zhou, Huilun Chen, Rongfang Yuan, Yuxin Liu, Yuefang Chen

**Affiliations:** School of Energy and Environmental Engineering, University of Science and Technology Beijing, Beijing, China

**Keywords:** sulfadiazine degradation, g-C3N4, photocatalysis, density functional theory, degradation pathway

## Abstract

Sulfadiazine (SDZ) is a common antibiotic pollutant in wastewater. Given that it poses a risk as an environmental pollutant, finding effective ways to treat it is important. In this paper, the composite catalytic material g-C_3_N_4_/Ag/γ-FeOOH was prepared, and its degradation performance was studied. g-C_3_N_4_/Ag/γ-FeOOH had a superior degradation effect on SDZ than g-C_3_N_4_ and γ-FeOOH. Compared with different g-C_3_N_4_ loadings and different catalyst dosages (5, 10, 25, and 50 mg/L), 2 mg/L g-C_3_N_4_/Ag/γ-FeOOH with a g-C_3_N_4_ loading of 5.0 wt% has the highest degradation promotion rate for SDZ, reaching up to 258.75% at 600 min. In addition, the photocatalytic enhancement mechanism of the catalyst was studied. Density functional theory (DFT) calculations indicated that the enhancement of photocatalytic activity was related to the narrowing of the forbidden band and the local electron density of the valence band. The bandgap of the catalyst was gradually narrowed from 2.7 to 1.05 eV, which can increase the light absorption intensity and expand the absorption edge. The density of states diagram showed that the local resonance at the interface could effectively improve the separation efficiency of e^−^-h^+^ pairs. Four degradation paths of SDZ were speculated based on DFT calculations. The analysis confirmed that the degradation path of SDZ primarily included Smiles-type rearrangement, SO_2_ extrusion, and S-N bond cleavage processes.

## Introduction

Sulfonamides (SAs) as a broad spectrum of drugs play an important role in protecting human health, but they pose risks as environmental pollutants because of their extensive use and widespread occurrence in the environment. According to a previous study, the livestock industry used nearly 6000 tons of SAs in 2013, of which SDZ accounted for more than 15% (Zhang et al., 2015). Pharmaceutical wastewater containing sulfadiazine (SDZ) is a new class of environmental pollutants that has been discharged into rivers and lakes (Duan et al., 2020). Fourteen SAs were detected in the surface water of 8 typical lakes in China, and the mass concentration ranged from not detected (ND) to 940 ng/L (Liu et al., 2018). Long-term exposure to SDZ may cause disease in humans (Pouretedal and Sadegh, 2014; Wen et al., 2017; Isawi, 2019), and more importantly, bacteria exposed to antibiotics for long periods may become resistant (Patel et al., 2019), decreasing the efficacy of antibiotics in clinical treatments. In addition, SDZ undergoes complex chemical reactions upon entering the environment, including extremely complex degradation and transformation (Kümmerer, 2009a; Kümmerer, 2009b); the intermediate products are also different (Patel et al., 2019). With the large-scale use of SDZ, its migration, degradation, transformation mechanism, and potential ecological risks have received considerable attention.

Photocatalysis is an advanced oxidation process that can produce reactive oxygen species (ROS) during the reaction process, which can mineralize most of the organic matter into CO_2_ and H_2_O. It has been widely used in wastewater treatment for its high efficiency and environmentally friendly properties (Zhang et al., 2009; Viet et al., 2019; Dong et al., 2020). The main problem of photocatalysis technology is how to improve the performance of the catalyst. Although some photocatalytic materials such as TiO_2_ (Ibukun et al., 2019), ZnO (Rodrigues et al., 2020), CdS (Lei et al., 2019), ZrO_2_ (Siwińska-Ciesielczyk et al., 2020), and WO_3_ (Ghenaatgar et al., 2019) have been commonly used, their wide bandgap width, easy recombination of photoelectron-hole pairs, low quantum efficiency, and poor stability (Hernández-Alonso et al., 2009; Bethi et al., 2016; Hu et al., 2016) result in low photocatalytic efficiency. Among photocatalysts, g-C_3_N_4_ has emerged as a robust semiconductor photocatalytic material because it is a non-metallic element; has a narrow band gap width (2.70 eV),and stable chemical properties; and is less costly (Niu et al., 2012; Cao and Yu, 2014; Ye et al., 2015; Wei et al., 2019; Prasad et al., 2020). However, the small specific surface area of g-C_3_N_4_ and easy recombination of photo-generated carriers greatly reduce photocatalytic efficiency (Wen et al., 2017). To improve its photocatalytic efficiency, g-C_3_N_4_ has often been doped with materials, such as various elements (e.g., S, Pt, and Ag) (Ma et al., 2012; Wang et al., 2015; Xiao et al., 2016), semiconductors (e.g., Ag_3_PO_4_) (He et al., 2015a), carbon materials (e.g., graphene, etc.) (Liao et al., 2012), and dye sensitization (Ge et al., 2012); alternatively, heterojunction structures with metal oxides (e.g., FeOOH and ZnO) (He et al., 2015b) have been built.

The combination of g-C_3_N_4_ and Fe and its metal oxides used in photocatalytic degradation can have excellent degradation effects. The main iron oxides include goethite (R-FeOOH), lepidocrocite (γ-FeOOH), maghemite (γ-Fe_2_O_3_), and hematite (R-Fe_2_O_3_) with band gaps (Eg) of 2.10, 2.06, 2.03, and 2.02 eV, respectively. The slow conversion rate from Fe^3+^ to Fe^2+^ of the Fe material alone during the degradation of pollutants precludes the circulation of Fe ions and causes the loss of Fe. After Fe is combined with g-C_3_N_4_, g-C_3_N_4_ generates photogenerated electrons and holes under light conditions. The electrons can reduce Fe^3+^ to Fe^2+^ because of its strong reducibility, accelerate the cycle of Fe ions, and promote the migration and separation of electron-hole pairs. Several studies have examined photocatalytic degradation by the composite of g-C_3_N_4_ and iron materials (Song et al., 2018). For example, g-C_3_N_4_, ZnO, and Fe_2_O_3_ were combined to prepare heterogeneous photocatalysts with a degradation rate that was approximately 3 and 2.4 times higher compared with pure g-C_3_N_4_ and ZnO/g-C_3_N_4_, respectively (Di et al., 2019). Although the modification of g-C_3_N_4_ by doping other elements and compounds has improved the photocatalytic performance, few studies have examined the mechanism by which photocatalytic efficiency is enhanced after combining different materials with g-C_3_N_4_. Furthermore, few experiments have systematically analyzed the crystal structure, electronic structure, and optical properties of composite g-C_3_N_4_.

Density functional theory (DFT) calculations can theoretically explain the mechanism of photocatalytic enhancement. For example, determination of the energy band structure and electron transfer of g-C_3_N_4_/CdS composite catalyst revealed that the band gaps of g-C_3_N_4_, CdS, and g-C_3_N_4_/CdS gradually narrowed to 2.76, 2.36, and 2.02 eV, respectively. In addition, g-C_3_N_4_ and CdS would form a type-II heterojunction, and the internal electric field formed would inhibit the recombination of photo-generated carriers (Liu, 2015). Xiong (Xiong et al., 2016) also used DFT calculations to show that K-doped g-C_3_N_4_ can promote the migration and separation of photo-generated carriers effectively and that the catalyst had stronger photocatalytic activity compared with Na-doped g-C_3_N_4_.

In this study, the γ-FeOOH/g-C_3_N_4_ system was constructed. To accelerate the migration rate of electrons from g-C_3_N_4_ to γ-FeOOH, silver ions were introduced on γ-FeOOH/g-C_3_N_4_. The effect of the metal conductor Ag can promote the separation of photogenerated electron pairs and inhibit the recombination of electron pairs, the Ag loading improved the response range of g-C_3_N_4_ to visible light and increased the photocatalytic performance (Deng et al., 2021). The use of Ag-based catalysts such as Ag_3_PO_4_ (Chen et al., 2017), Ag_2_CrO_4_ (Rajalakshmi et al., 2021) for photocatalytic degradation has been widely applied. It is reported (He et al., 2017a; Song et al., 2018) that Ag can increase the electron transfer rate between components in the composite material and that its surface plasmon resonance can permit the photocatalyst to strongly absorb visible light. Furthermore, it has been reported that the combination of g-C_3_N_4_, γ-FeOOH, and Ag to form g-C_3_N_4_/Ag/γ-FeOOH catalytic materials has a high degradation rate of the azo dye methyl orange under visible light (He et al., 2017a).

We thus prepared a composite catalyst g-C_3_N_4_/Ag/γ-FeOOH and investigated the effects of different doping amounts of g-C_3_N_4_ during SDZ degradation. The photocatalytic enhancement mechanism, as well as changes in the DOS and band structure before and after doping Ag/γ-FeOOH in g-C_3_N_4_ was studied. In addition, the degradation pathway of SDZ was inferred based on the results of LC-MS detection, and DFT calculations were used to provide theoretical support.

## Materials and Methods

### Materials

Sulfadiazine (SDZ, C_10_H_10_O_2_N_4_S, 99%) was purchased from Sigma-Aldrich, United States. Iron chloride tetrahydrate (FeCl_2_·H_2_O), sorbic acid (C_6_H_8_O_2_), and sodium hydroxide (NaOH) were obtained from Beijing Chemical Co., China. Suwannee River Humic Acid (SRHA) was obtained from the International Humic Substances Society. Isopropyl alcohol (C_3_H_8_O) was purchased from Tianjin Fuchen Chemical Reagent Co., China. All other chemicals and reagents were analytical grade or higher.

### Preparation of Catalysts

In the synthesis process, 7 g urea and 3 g melamine were calcined at 550°C for 4 h at a heating rate of 5°C per minute, cooled, ground into a fine size, and kept at 550°C for 2 h. In the synthesis process, 11.93 g (FeCl_2_·4H_2_O) was dissolved in 300 ml of deionized water and filtered. Next, 200 ml of 0.5 mol/L NaOH solution was added to the filtrate to adjust the pH to 6.8, followed by thorough stirring to ensure that the reaction with the filtrate was complete. The resultant solution was then centrifuged at 25°C with a rotating speed of 4500 r/min for 25 min. The solution was thoroughly cleaned with deionized water until the supernatant was completely clear, and the supernatant was removed. The remaining solid material was dried at 60°C for 10 h to obtain γ-FeOOH.

Details of the g-C_3_N_4_/Ag/γ-FeOOH synthesis methods are provided in the (Supplementary Text S1).

### Catalysts Characterization

X-ray crystallographic data were obtained by X-ray diffraction with Cu/Kα radiation (λ = 1.5418 Å). The morphology, particle size, and elemental composition of the samples were assessed using a field emission scanning electron microscope (FESEM, Mira 3-XMU) and energy dispersive spectroscopy (EDS). The molecular structure of C_3_N_4_ catalysts was determined by Raman spectroscopy (Jobin Yvon HR800). The chemical composition of the catalyst was analyzed by X-ray photoelectron spectroscopy (RuiYing Xpert Pro MPD). The Fourier transform infrared (IR) spectra of the samples were recorded by an infrared spectrometer (Nexus 410) to characterize the surface functional groups of g-C_3_N_4_, γ-FeOOH, and g-C_3_N_4_/Ag/γ-FeOOH.

### Photodegradation Procedures

All experiments were conducted in a multifunctional photochemical reactor (Shanghai Yanzheng YZ-GHX-A type) (Supplementary Figure S1). A 500-W xenon lamp was used as the light source, and 40 ml of 5 mg/L SDZ reaction solution was placed into a quartz reaction tube with different additives. During the experiment, the temperature of the reaction environment was controlled by circulating condensate and an air cooling system (25°C). Sampling was performed at 0, 30, 60, 120, 240, 360, and 600 min, and 500 μl was removed at each sampling event for concentration measurements.

### Sulfadiazine Concentration Determination

The concentration of SDZ was determined by liquid chromatography (LC-20AD) with a reverse C18 column (5 μm, 4.6 mm × 150 mm). The mobile phase was acetonitrile/(water+ 0.1% acetic acid) with a volume ratio of 40/60 and a flow rate of 1 ml/min. The UV detector wavelength was 265 nm, the temperature was 30°C, the injection volume was 20.0 µl, the peak time was approximately 4.5 min, and the peak area was quantified by the external standard method. The samples had to be filtered through a 0.22-μm filter membrane before HPLC determination to avoid blocking the instrument pipeline.

### Density Functional Theory Calculation

The first principle constants were calculated using the Vienna Ab initio Simulation Package and the projected amplified wave method based on DFT. The exchange functional was treated using the generalized gradient approximation of Perdew-Burke-Ernzerhof functional (Perdew et al., 1996). The cutoff energy for electronic wave functions was set to 400 eV. For bulk structure, k-points were sampled in a 4 × 4 × 4 Monkhorst−Pack grid. In addition, the role of the van der Waals force was considered in the calculation. The self-consistent calculations applied a convergence energy threshold of 10^−6^ eV. Geometry relaxations were conducted until the residual forces on each ion were smaller than 0.05 eV/Å. The vacuum spacing was at least 10 Å in the direction perpendicular to the catalyst plane. The Brillouin zone integration was performed using 3 × 3 × 1 Monkhorst–Pack K-point sampling for the surface and interface. In addition, spin polarization was also considered in all calculations. The Hubbard U (DFT + U) correction for 3D transition metals was set based on previous studies (Gong et al., 2019).

The free energy was calculated using the equation (No̸rskov et al., 2006):

G=E+ZPE−TS

where G, E, ZPE, and TS are the free energy, total energy from DFT calculation, zero-point energy, and entropic contributions (T was set to 300 K), respectively. ZPE could be derived after frequency calculation by (Nørskov et al., 2004):

$$ZPE=1/2\sum hv_{i}$$

Furthermore, the TS values of adsorbed species are calculated after obtaining the vibrational frequencies (Bendavid and Carter, 2013):

$$TS_{v}=K_{B}T\left[\underset{K}{\sum }ln\left(1/1-exp\left(-hv/K_{B}T\right)\right)+\underset{K}{\sum }\text{hv}/K_{B}T\cdot exp\left(hv/K_{B}T-1\right)\right]$$

## Results and Discussion

### Characterization of Catalysts

**FIGURE 1:**
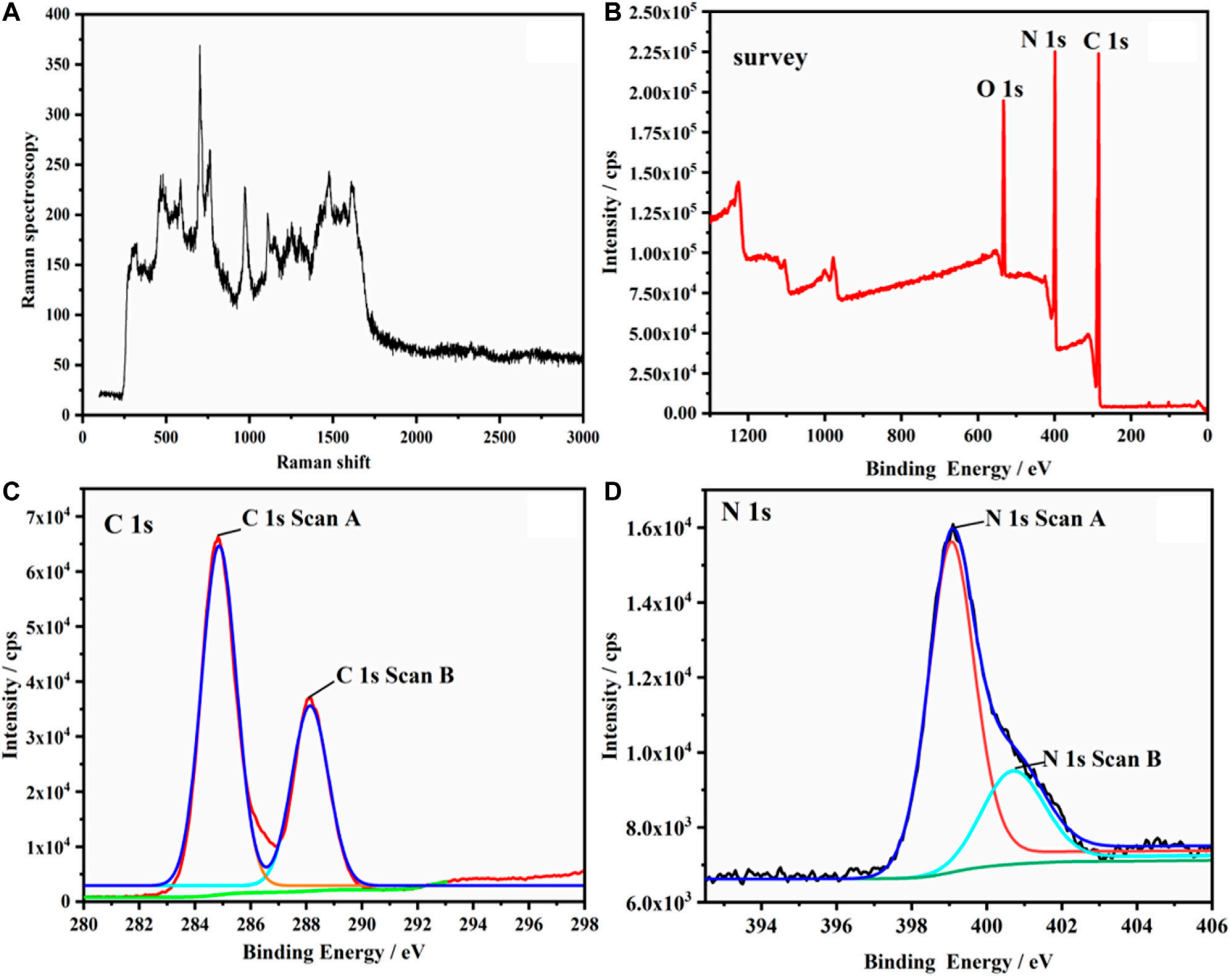
**(A)** Raman spectra of g-C_3_N_4_, **(B)** XPS full spectrum of g-C_3_N_4_, **(C)** XPS C spectrum of g-C_3_N_4_, **(D)** XPS N spectrum of g-C_3_N_4_.

Figure 1 shows the Raman spectra, XPS full spectrum, and C and N spectra of g-C_3_N_4_. Pure g-C_3_N_4_ was mainly composed of three elements: C, N, and O. O1s may be attributed to the surface adsorption (Figures 1A,B). Figure 1C was divided into two binding energies 287.6 and 284.6 eV after peak fitting. The former was attributed to the thiazine structure N=C-N group, and the latter was assigned to sp^2^-bonded C in the aromatic ring (C-C bonding). The two peaks of binding energy (398.6 and 400.4 eV) in Figure 1D correspond to the C-N-H bond and C-N-H bond, respectively (He et al., 2017b).

**FIGURE 2:**
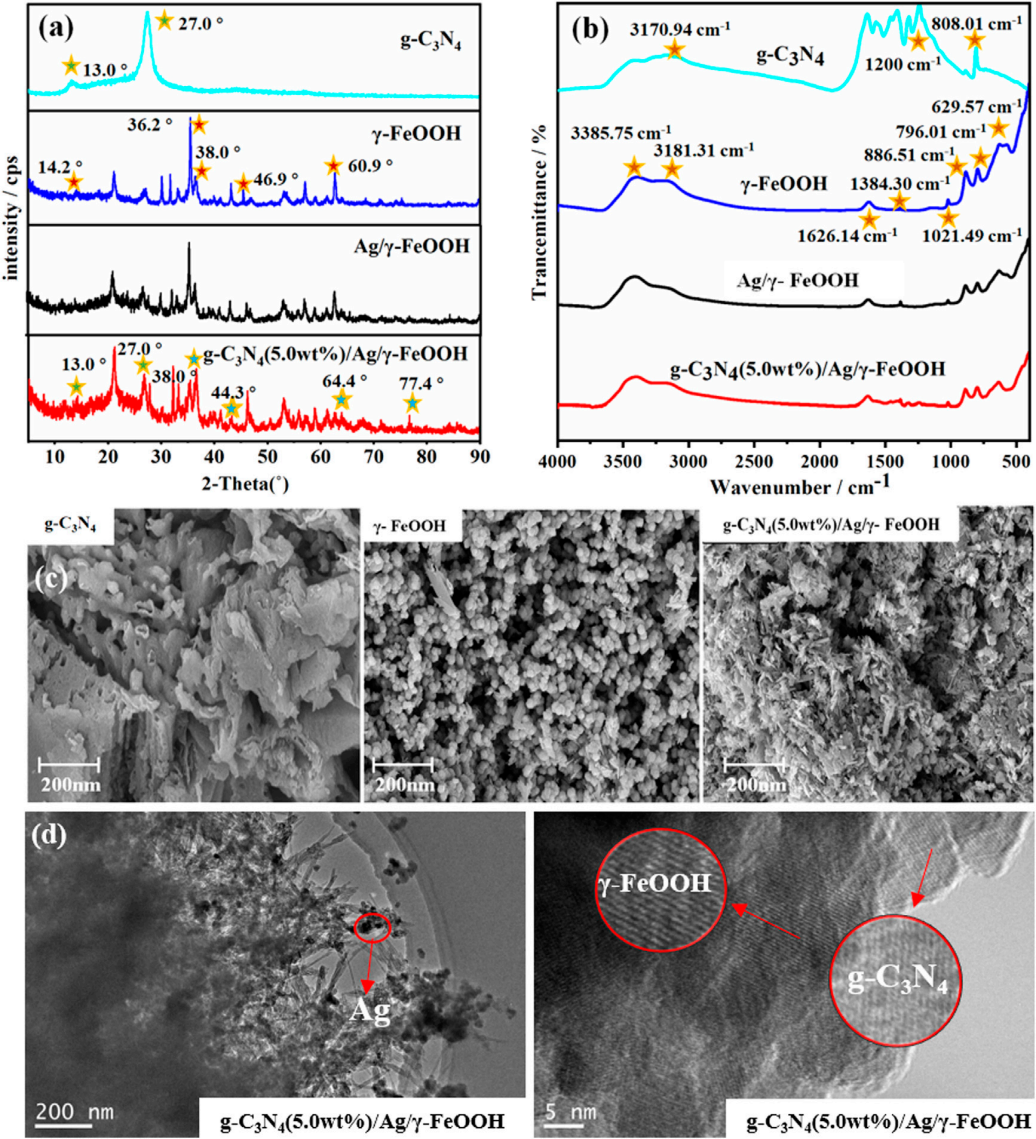
**(A)** XRD patterns of the catalysts, **(B)** FT-IR spectrograms of the catalysts, **(C)** SEM of g-C_3_N_4_, γ-FeOOH and g-C_3_N_4_(5 wt%)/Ag/γ-FeOOH, **(D)** HRTEM characterization of g-C_3_N_4_(5 wt%)/Ag/γ-FeOOH.

The XRD patterns of γ-FeOOH, γ-FeOOH/Ag, and g-C_3_N_4_ (5.0 wt%)/Ag/γ-FeOOH are shown in Figure 2A. For γ-FeOOH, the peaks at 2θ values of 14.2, 36.2, 38.0, 46.9, and 60.3 were lepidocrocite (He et al., 2017a), which corresponded to the (200), (301), (111), (020), and (002) lepidocrocite crystal faces, respectively. The characteristic diffraction peaks of Ag appeared in g-C_3_N_4_/Ag/γ-FeOOH, XRD diffraction peaks at 2θ values of 38°, 44.3°, 64.4°, 77.4°, matched well with the Ag standard pattern (JCP-DS No.04-0783) (Figure 2A), and can be assigned to (111), (200), (220), and (311) crystal plane (Deng et al., 2021). Compared with pure g-C_3_N_4_, the intensity of the (002) peak in the g-C_3_N_4_/Ag/γ-FeOOH diffraction peak was reduced and slightly offset. The introduction of Ag destroyed the thiazine structure unit of g-C_3_N_4_, due to the different ionic radii of Ag, C, and N. It is indicated that Ag successfully attached to the surface of γ-FeOOH. The spectral lines of γ-FeOOH and its composite catalyst samples had similar peaks. Therefore, the surface structure of γ-FeOOH was not changed after the addition of g-C_3_N_4_ and Ag. The FT-IR spectra are shown in Figure 2B. For g-C_3_N_4_, the peak at 808.01 cm^−1^ can be attributed to the characteristic breathing pattern of the thiazine unit (Nørskov et al., 2004), and a series of peaks between 1,200 and 700 cm^−1^ were attributed to the typical stretching modes of C/N heterocycles. The peak at 3170.94 cm^−1^ was attributed to the N-H and O-H stretching vibrations of physically adsorbed water. The γ-FeOOH in-plane peaks were at 1384.30, 1626.14, and 1021.49 cm^−1^, and the out-of-plane Fe-O-H bending vibrations were at 629.57, 796.01, and 886.51 cm^−1^. The peaks at 3181.31 and 3387.75 cm^−1^ were primarily driven by the crystal H_2_O vibration. The IR spectrum of g-C_3_N_4_/Ag/γ-FeOOH included the peaks of pure γ-FeOOH and g-C_3_N_4_, indicating that both g-C_3_N_4_ and γ-FeOOH were present in the synthesized composite material.

The SEM image of the g-C_3_N_4_ revealed an obvious block structure and void structure (Figure 2C); furthermore, the size was uneven, and the surface was smooth. Pure γ-FeOOH was composed of spindle-shaped and spherical homogeneous particles, which were agglomerated into many pieces by relatively small particles. As we can see from the SEM image of g-C_3_N_4_/Ag/γ-FeOOH, after the growth of γ-FeOOH, the surface of the hybrid was rougher and thicker than pure g-C_3_N_4_. Compared with pure γ-FeOOH, the fiber bundle disappeared, indicating that g-C_3_N_4_ was dispersed by ultrasonic treatment and g-C_3_N_4_ was successfully loaded on the surface of γ-FeOOH. Observing the HRTEM image, we can find the presence of Ag, γ-FeOOH and g-C_3_N_4_ (Figure 2D). Ag and γ-FeOOH were coated with g-C_3_N_4_. It can be clearly seen the parallel and ordered lattice fringes of γ-FeOOH and g-C_3_N_4_, The lattice spacing is 0.269 and 0.334 nm, respectively, corresponding to the (130) and (002) crystal faces of γ-FeOOH and g-C_3_N_4_, which indicated the successful recombination of the three materials. The surface scan electronic image, mapping diagram, and total spectrum of the composite material g-C_3_N_4_ (2.5 wt%)/Ag/γ-FeOOH and g-C_3_N_4_ (5.0 wt%)/Ag/γ-FeOOH at a certain position are shown in Supplementary Figure S2. It can be found that the main constituent element Fe in the synthetic material g-C_3_N_4_(2.5 wt%)/Ag/γ-FeOOH accounted for a very large proportion, for 83.1%. The content of C (12.6%) was the second, followed by Ag (4.6%) element. Although the content of N could not be estimated, bright spots could be observed by the surface-scanned diagram, suggesting that the synthetic materials primarily included Fe, C, Ag, and N. The composition and proportion of g-C_3_N_4_ (5.0 wt%)/Ag/γ-FeOOH were similar to those when the loading amount of C_3_N_4_ was 2.5 wt%, the proportion of Fe element was reduced to 81%, but it was still the main element. The content of C element has increased by 1.8%, which was consistent with the increase in C_3_N_4_ loading.

**FIGURE 3:**
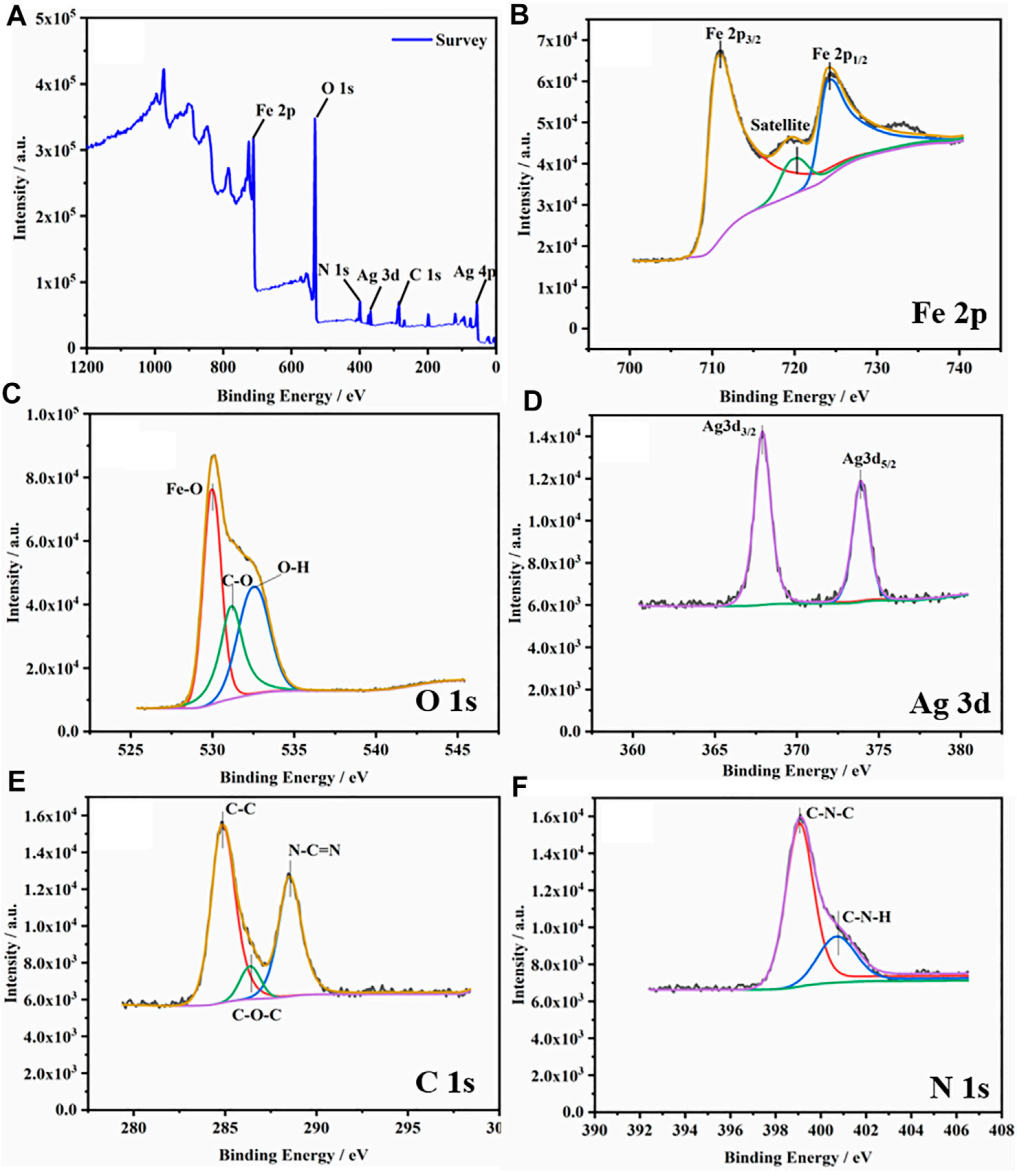
**(A)** XPS Spectra of g-C_3_N_4_(5 wt%)/Ag/γ-FeOOH. **(B)** High-resolution spectrum of Fe 2p in the composite, **(C)** O 1s, **(D)** Ag 3d, **(E)** C 1s, **(F)** N 1s.

XPS was used to further characterize the chemical composition and state of the composite material (Figure 3). XPS full spectrum of the g-C_3_N_4_/Ag/γ-FeOOH confirmed the presence of Fe, C, N, O, and Ag elements, of which the content of O was relatively high. Figure 3B shows the high-resolution spectrum of Fe 2p in the composite. Two well-resolved peaks are located at 710.9 and 724.0 eV, belonging to Fe 2p_3/2_ and Fe 2p_1/2_, respectively. The Fe 2p_3/2_ peak has an associated satellite peak at 719.7 eV. The satellite peak is the result of charge transfer shielding, which is related to the presence of Fe^3+^ in γ-FeOOH (Zhang et al., 2018a; Liu et al., 2016). The characteristic peaks with binding energies at 529.9 and 532.5 eV in Figure 3C can be attributed to the Fe-O bond and OH bond in γ-FeOOH (Chemelewski et al., 2014), respectively. A characteristic peak appears at 531.1eV, which may be attributed to C=O. As shown in Figure 3D, typical peaks of Ag 3d can be found. The peaks at 367.9 and 373.9 eV belong to Ag 3d_3/2_ (Ag^+^) and Ag 3d_5/2_ (Ag^+^). The results are consistent with previous reports (Chen et al., 2013). The C 1s spectrum of g-C_3_N_4_/Ag/γ-FeOOH is similar to the XPS of pure g-C_3_N_4_. There are two main peaks at 284.8 and 287.8 eV that can be assigned to the C-C and N-C=N coordination, respectively. The peak with a binding energy of 286.3 eV is considered to be a surface hydroxyl bond (C-OH) (Yan et al., 2016). The two peaks of binding energy 399.0 and 400.6 eV in Figure 3F correspond to the C-N-C bond and C-N-H bond (Cao et al., 2017), respectively.

**FIGURE 4:**
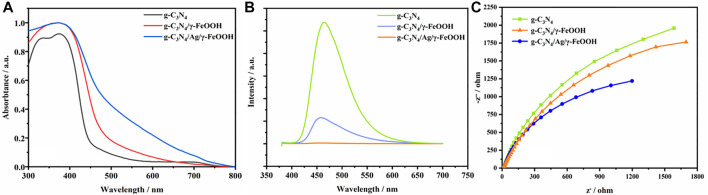
**(A)** UV−vis absorption spectra of g-C_3_N_4_, g-C_3_N_4_/γ-FeOOH, g-C_3_N_4_/Ag/γ-FeOOH, **(B)** Photoluminescence spectra of pure g-C_3_N_4_, g-C_3_N_4_/γ-FeOOH, g-C_3_N_4_/Ag/γ-FeOOH, **(C)** Electrochemical impedance spectroscopy of g-C_3_N_4_, g-C_3_N_4_/γ-FeOOH, g-C_3_N_4_/Ag/γ-FeOOH.

Optical properties determine the ability of composite materials to absorb and utilize light. It can be observed from the ultraviolet-visible diffuse (UV-vis) reflectance spectrum that the light absorption capacity of g-C_3_N_4_/Ag/γ-FeOOH is significantly higher than g-C_3_N_4_, g-C_3_N_4_/γ-FeOOH (Figure 4A). The absorption edge of pure g-C_3_N_4_ starts at 450 nm. The absorption edge of the composite material has a red shift, which means a broadening of the visible light response range (Oh et al., 2017; Fu et al., 2018), indicating that it has a strong ability to absorb visible light after being composited with γ-FeOOH. Meanwhile, the introduction of Ag helped to enhance the light absorption of the material in the range of 450–800 nm and increase the light response intensity of the material in the visible light range. Owe to the surface plasmon resonance of Ag, the absorption of light by the Ag-containing sample is stronger than that without Ag, which promotes the generation of photogenerated carriers and the surface photocatalytic reaction.

Photoluminescence (PL) spectroscopy is an effective means to characterize electron migration and separation efficiency in composite photocatalytic materials. Therefore, we performed PL analysis on g-C_3_N_4_, g-C_3_N_4_/γ-FeOOH, g-C_3_N_4_/Ag/γ-FeOOH. As shown in Figure 4B, for g-C_3_N_4_, electron-hole pairs are easily recombined due to the presence of a π-bonded polymer structure, and therefore have a photoluminescence phenomenon. The characteristic peak of its luminescence is centered at 465 nm and the emission band from 400 to 550 nm. After recombination with γ-FeOOH, electrons migrate from g-C_3_N_4_ to γ-FeOOH, and the characteristic luminescence peak is weakened, which indicates that the recombination of g-C_3_N_4_ and γ-FeOOH promote the migration and separation of photogenerate carriers. The emission peak disappeared after Ag was compounded, indicating that Ag was successfully loaded on the surface of γ-FeOOH and g-C_3_N_4_. Ag could act as a bridge for electron transfer between g-C_3_N_4_ and γ-FeOOH, and promoted the migration of photogenerated electrons to γ-FeOOH. Electrochemical impedance spectroscopy (EIS) is an electrochemical method for studying interface resistance and charge separation. The resistance value of different materials can be obtained by comparing the diameter of a semicircle when illuminated. It can be seen from Figure 4C that the semi-circular arc of g-C_3_N_4_/Ag/γ-FeOOH is smaller than g-C_3_N_4_ and g-C_3_N_4_/γ-FeOOH, indicating that the g-C_3_N_4_/Ag/γ-FeOOH have the smallest impedance value, and g-C_3_N_4_/Ag/γ-FeOOH is more conducive to the transmission of photogenerated electrons.

The above characterization and energy band analysis indicates that the composite material is beneficial to reduce the contact barrier of the interface while enhancing the electronic coupling effect of the semiconductor, and meanwhile, it is beneficial to the formation of more photo-generated electron-hole pairs, thus achieving the purpose of improving the photocatalytic performance of g-C_3_N_4_ (Zhang et al., 2018b). We infer that when g-C_3_N_4_ is excited, photo-generated carriers transition from g-C_3_N_4_ to γ-FeOOH and migrate out, thus realizing the effective migration and separation of photo-generated carriers. After Ag is compounded, it can serve as a bridge for electron transfer between g-C_3_N_4_ and γ-FeOOH, further promoting the migration of photo-generated electrons to γ-FeOOH, and the plasmon resonance on the Ag surface further promoted the absorption of visible light, which will provide more hole oxidizer to improve the efficiency of photocatalytic degradation of pollutants.

### Photocatalytic Degradation of Sulfadiazine

#### Catalytic Degradation of Sulfadiazine by g-C_3_N_4_ and γ-FeOOH

**FIGURE 5:**
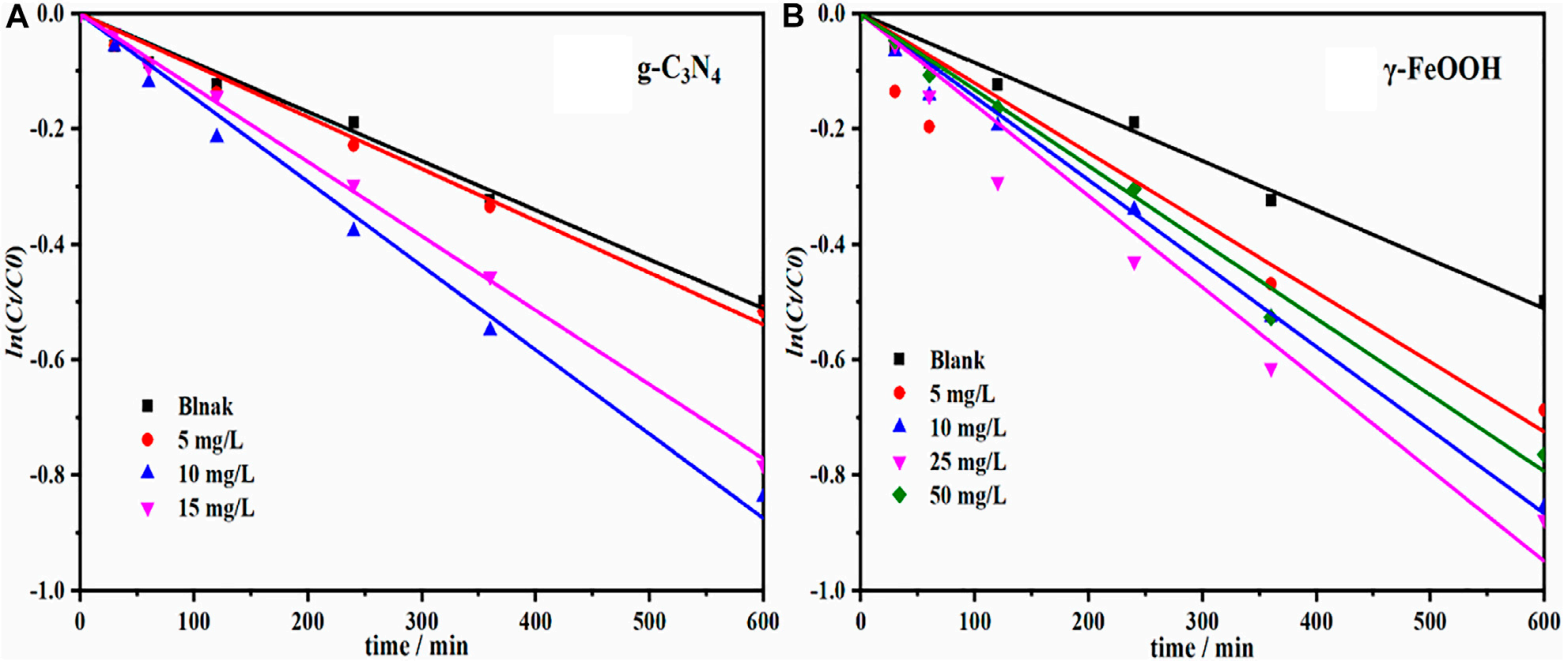
**(A)** Catalytic degradation of SDZ by g-C_3_N_4_ (The concentrations of g-C_3_N_4_ were 0, 5, 10, 15, respectively) and **(B)** γ-FeOOH (The concentrations of γ-FeOOH were 0, 5, 10, 25, 30, respectively).

Figure 5A shows the effect of different concentrations of g-C_3_N_4_ on the SDZ photocatalysis rate. The kinetic equations and parameters for SDZ degradation are listed in Supplementary Table S1. The promotion effect of the catalyst is expressed by the promotion rate (*ζ*):

$$\zeta =\left(k_{catalyst\;}-\;k_{blank}\right)/k_{blank}$$ (1)

Compared with natural SDZ photocatalysis, the SDZ photocatalysis promotion rate reached 73.75% under 10 mg/L g-C_3_N_4_ catalysis; when the g-C_3_N_4_ concentration was increased to 15 mg/L, the SDZ photocatalysis rate constant *k* decreased, but the promotion rate was still 61.25%.

γ-FeOOH also had a certain catalytic effect on the photocatalysis of SDZ (Figure 3C and Supplementary Table S2). When the γ-FeOOH concentration was 5, 10, 25, and 50 mg/L, the reaction promotion rates were 12.5, 28.75, 71.25, and 55%, respectively. The SDZ photocatalysis rate constant *k* was 1.37 × 10^−3^ min^−1^, and the reaction rate was increased by 71.25%.

#### Catalytic Degradation of Sulfadiazine by g-C_3_N_4_/Ag/γ-FeOOH

**FIGURE 6:**
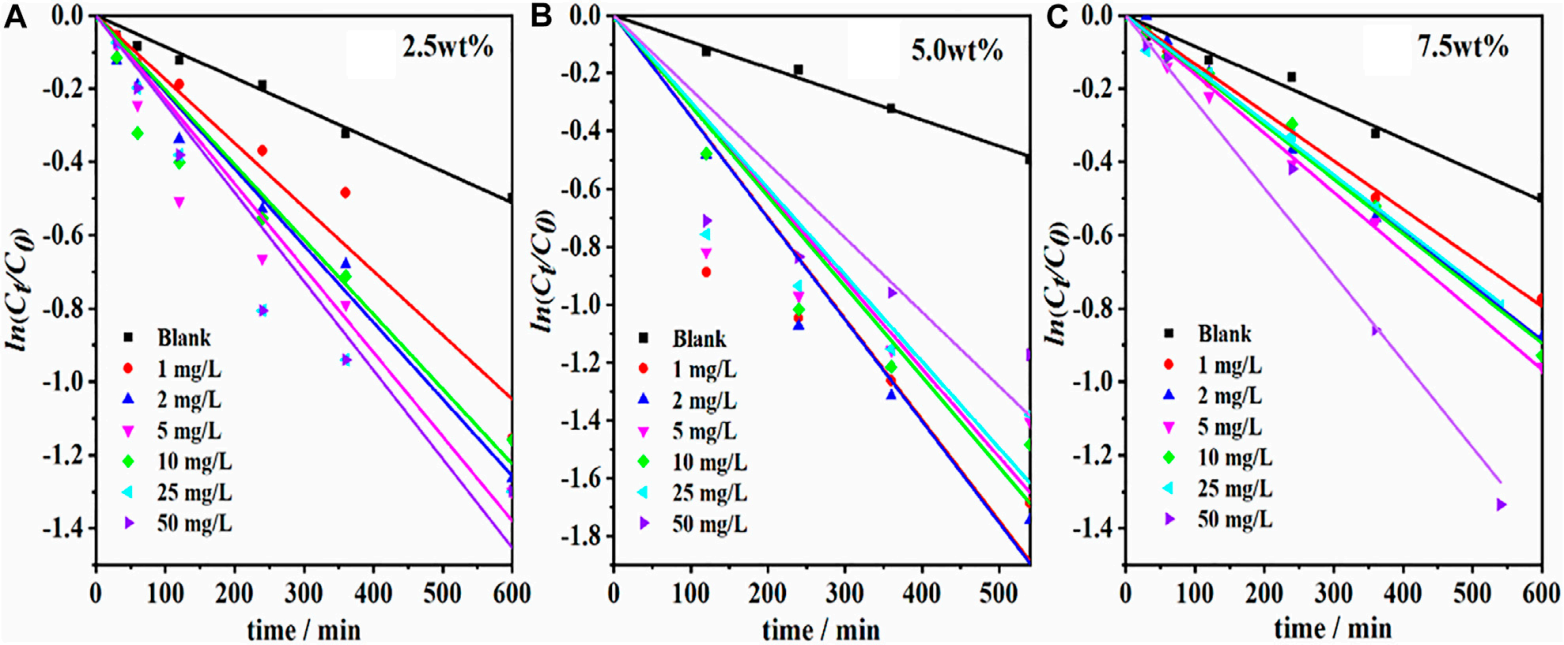
Degradation of SDZ by different g-C_3_N_4_ loading (**(A)** 2.5% wt, **(B)** 5.0% wt, **(C)** 7.5% wt) and different concentration g-C_3_N_4_/Ag/γ-FeOOH (0, 1, 2, 5, 10, 25, 50 mg/L).

The photodegradation of SDZ under different concentrations of g-C_3_N_4_/Ag/γ-FeOOH is shown in Figure 6, and the results are shown in Supplementary Table S3. The g-C_3_N_4_(2.5 wt%)/Ag/γ-FeOOH promoted the catalytic effect (Figure 6A and Supplementary Table S3) compared with the blank sample. Furthermore, 1 mg/L g-C_3_N_4_(2.5 wt%, 5 wt%, 7.5 wt%)/Ag/γ-FeOOH promoted the SDZ photocatalysis reaction rate to 135.00, 138.75, and 57.5%, respectively. It indicate that complex Ag or g-C_3_N_4_ to γ-FeOOH can increase the degradation rate of SDZ. Notably, g-C_3_N_4_/Ag/γ-FeOOH showed better photocatalytic activity than pure g-C_3_N_4_ and γ-FeOOH mainly due to the surface plasmon resonance of Ag, which can increase the absorption of visible light. When the g-C_3_N_4_ loading was 7.5%, the degradation promotion rate decreased significantly. The reasons may be as follows: as the g-C_3_N_4_ content continuously increase, on the one hand, it covered a large number of mesoporous and microporous structures on the surface of Ag/γ-FeOOH, and weakened the adsorption capacity of Ag/γ-FeOOH on organic pollutants; On the other hand, it also reduced the uniformity of the dispersion of g-C_3_N_4_ particles on the Ag/γ-FeOOH surface, and more g-C_3_N_4_ particles appeared agglomeration, Hence, the agglomerated g-C_3_N_4_ accelerated the recombination rate of photo-generated electron-hole on the catalyst surface, to further inhibit the effective separation of carriers, and then weakened the photocatalytic performance of the composite material (Ai et al., 2019). When the concentrations of g-C_3_N_4_(2.5 wt%)/Ag/γ-FeOOH were 2 mg/L and 5 mg/L, the reaction promotion rates were 141.25 and 125.0%, respectively. When the catalyst concentration increased again, the degradation rate remained at 60%. Under the catalysis of 2, 5, and 10 mg/L g-C_3_N_4_(5.0 wt%)/Ag/γ-FeOOH, the SDZ photocatalysis reaction promotion rates were 258.75, 76.25, and 183.75%, respectively. As the catalyst concentration increased to 25–50 mg/L, the maximum promotion effect was only 85%. Thus, once the catalyst dosage increased above a certain value, the degradation removal rate decreased for two reasons: 1) the increment of turbidity in the medium and 2) the increment in the agglomeration of the catalyst particles caused the scattering and shielding of UV (Wang et al., 2018). The concentrations of g-C_3_N_4_(7.5 wt%)/Ag/γ-FeOOH were 1, 5, and 10 mg/L, and the reaction promotion rates were 57.5, 95.0, and 85.0%, respectively. The increase in the promotion rate might stem from the fact that the composite catalyst absorbed more photons and generated more active species.

By comparison, g-C_3_N_4_(2.5 wt%)/Ag/γ-FeOOH had a superior catalytic effect at low concentrations than g-C_3_N_4_(7.5 wt%)/Ag/γ-FeOOH, but g-C_3_N_4_(7.5 wt%)/Ag/γ-FeOOH had better performance at high concentrations. At a concentration of 50 mg/L, the photocatalysis reaction rate to SDZ was 206.25%. However, compared with 50 mg/L g-C_3_N_4_(7.5 wt%)/Ag/γ-FeOOH and 2 mg/L g-C_3_N_4_(5.0 wt%)/Ag/γ-FeOOH under the same conditions, the latter had a better catalytic effect.

### Free Radical Distribution and Mechanism Analysis

**FIGURE 7:**
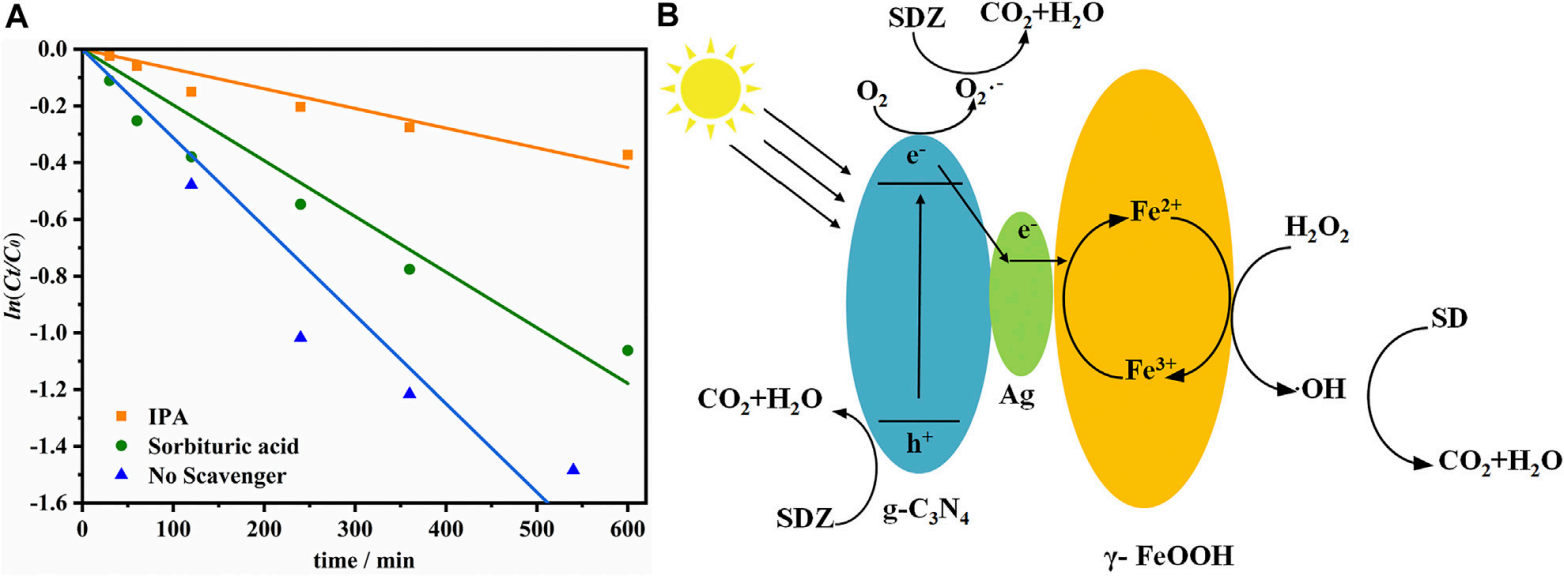
**(A)** The effect of adding different quenchers on the degradation of SDZ, **(B)** Mechanism of catalytic degradation of SDZ.

To determine whether ROS such as OH were produced and whether these ROS are key components during the photodissociation of SDZ. Free radical capture experiments were used to investigate the free radicals generated in the reaction system. IPA (·OH quenchers) and sorbituric acid (^3^DOM^*^ quenchers) were added to the reaction system (Figure 7A). Under the catalysis of 10 mg/L g-C_3_N_4_/Ag/γ-FeOOH, the photodissociation rate of SDZ significantly decreased after the addition of IPA. After adding sorbic acid, the degradation rate of SDZ only showed a small decrease. Thus, ·OH and ^3^DOM^*^ might have appeared in the photodissociation process and contribute to the degradation of SDZ. The contribution rate of OH was 69.60%. Similarly, the contribution rate of ^3^DOM^*^ was 13.66%. He et al. reported that after adding IPA and TEOA to the reaction system, the photodegradation efficiency of g-C_3_N_4_/Ag/γ-FeOOH was significantly reduced, indicating that OH and h^+^ are the main free radicals (He et al., 2017a).

The catalytic mechanism of g-C_3_N_4_/Ag/γ-FeOOH was the synergistic mechanism of photocatalysis and the heterogeneous Fenton process (). First, under visible light, the electrons generated by the valence band (VB) of g-C_3_N_4_ transitioned to the conduction band (CB), forming photogenerated electrons and holes. When g-C_3_N_4_ was recombined with γ-FeOOH, the visible light absorption capacity of the catalyst was enhanced. In addition, electrons can reduce O_2_ to O_2_^−^, which has a strong oxidation ability and can oxidize SDZ to CO_2_ and H_2_O. Second, Ag exhibited a localized surface plasmon resonance effect under visible light, resulting in a collective oscillation of conduction electrons interacting with electromagnetic radiation to produce unique optical properties. On the one hand, under the action of Ag, some photoelectrons flowed from CB of g-C_3_N_4_ to Ag, and eventually transferred to γ-FeOOH. The local surface plasmon resonance effect generated by the hot spot structure formed by Ag increased the generation rate of photogenerated e^−^-h^+^ pairs on the surface of g-C_3_N_4_, thereby improving the photocatalytic activity. It formed an electron trap on the surface of g-C_3_N_4_, which reduced the recombination probability of photoinduced e^−^ and h^+^. On the other hand, under the action of visible light, the Ag could effectively activate the O_2_, which would adsorb on the surface of the catalyst, to form strong oxidizing O_2_^−^, thus contributing to the degradation of SDZ (Ai et al., 2019; Deng et al., 2021). Third, the Fenton reaction took place. Fe^3+^ was converted to Fe^2+^ under the reduction of electrons to form a reusable heterogeneous Fenton cycle system. According to the discussion above, the heterogeneous Fenton-like photocatalytic process likely synergistically increased the degradation rate of SDZ. Photocatalysis can provide the electrons required by the Fenton process to maintain the Fe^2+^ concentration. Similarly, the heterogeneous Fenton-like process promoted photocatalysis by improving the catalyst’s absorption of visible light and promoting the migration and separation of photo-generated carriers.

### Density Functional Theory Calculation and Electronic Structure

DFT calculations were used to predict the crystal structure of g-C_3_N_4_ before and after recombination with Ag and γ-FeOOH. The optimized g-C_3_N_4_ single-layer planar structure was shown in Supplementary Figure S3A. The optimized g-C_3_N_4_ lattice parameter were a = b = 7.135, which is consistent with the experimental results and previous theoretical calculations. The heptazine structure of g-C_3_N_4_ belongs to an orthogonal system, and its space base is Cmc21 (Wang et al., 2009). γ-FeOOH belongs to the orthorhombic system, which is closely packed with γ-type cubes. The optimized lattice parameters of γ-FeOOH were a = 6.14 and b = 9.32 (Supplementary Figure S3B). In this study, the g-C_3_N_4_/γ-FeOOH structure was constructed by placing a γ-FeOOH octahedron on top of a single layer of g-C_3_N_4_. Thus, Ag was used to construct the composite catalyst g-C_3_N_4_/Ag/γ-FeOOH. The optimized structure of g-C_3_N_4_/Ag/γ-FeOOH is shown in Supplementary Figures S3C,D. At the g-C_3_N_4_/Ag/γ-FeOOH interface, the C-N bond lengths were about 1.34, 1.44, and 1.47 Å, and the Fe-O bond lengths were approximately 1.85 and 1.92 Å. O-H bond lengths were 0.97 Å. These values were slightly different from the C−N bond length (1.33, 1.39, and 1.47 Å) of the single-layer g-C_3_N_4_ nanoflake as well as the Fe-O bond length (2.00 and 2.12 Å) and O-H bond length (1.00 Å) of γ-FeOOH. Thus, the interaction of the three substances was weak, and van der Waals interaction occurred on the surfaces of the three substances.

**FIGURE 8:**
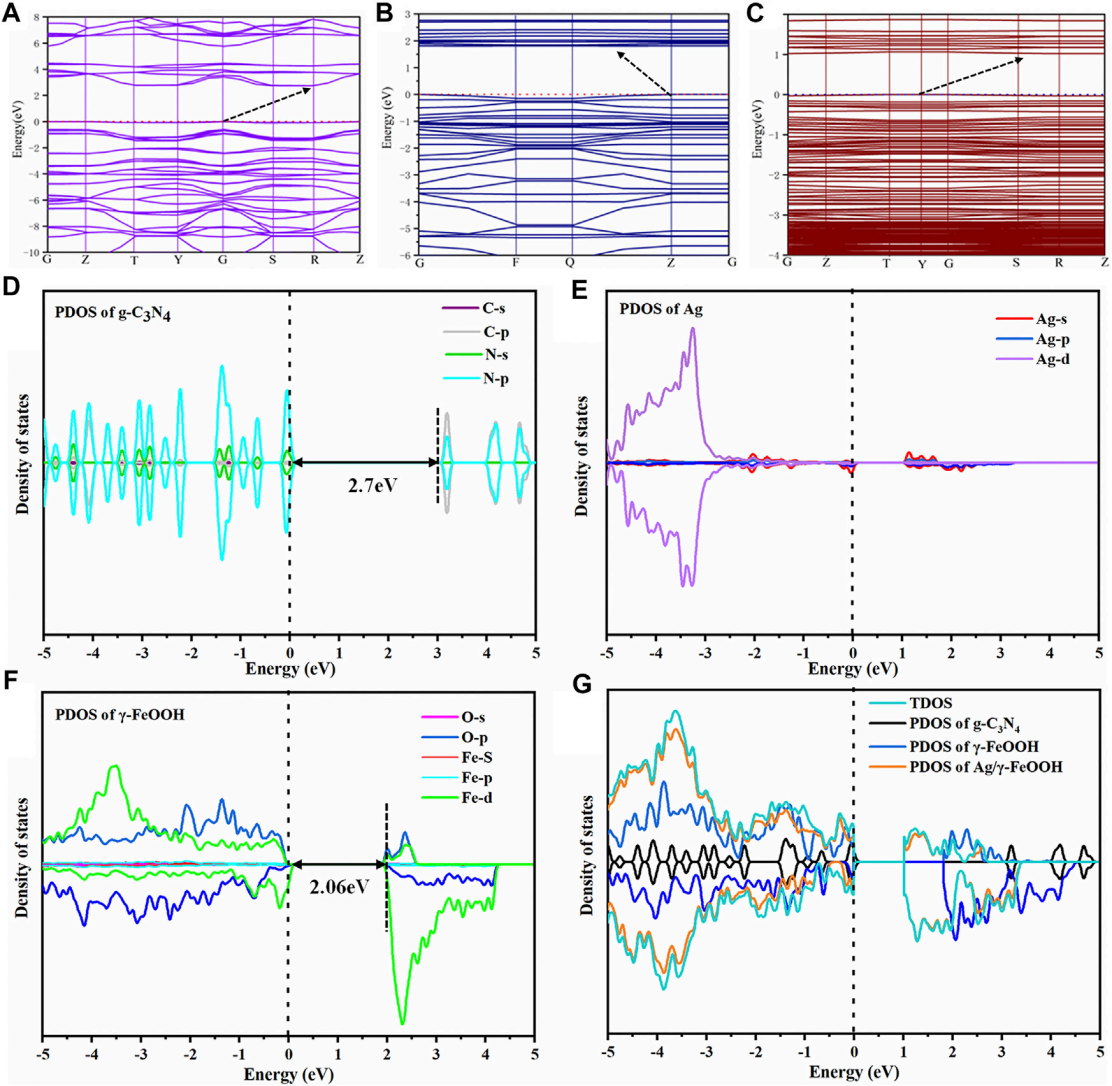
**(A)** The energy band structure of pure g-C_3_N_4_, **(B)** the energy band structure of g-C_3_N_4_/γ-FeOOH, **(C)** the energy band structure of g-C_3_N_4_/Ag/γ-FeOOH. **(D–G)** the PDOS and DOS of the catalysts.

The energy band structure, total density of state (TDOS), and project density of state (PDOS) of the catalysts were determined to characterize the high catalytic activity of the composite catalyst and reveal the photocatalytic enhancement mechanism. The valence band maximum (VBM) of pure g-C_3_N_4_ was located at point G, and the conduction band minimum (CBM) was located at point R (Figure 8A), which indicated that g-C_3_N_4_ was an indirect bandgap semiconductor. The calculated band gap was 2.7 eV, which is consistent with the experimental value and previous calculations (Wang et al., 2009). The VBM of pure g-C_3_N_4_ was primarily composed of N 2p orbitals. In contrast, the CBM was primarily composed of C 2p orbitals and only a small portion of N 2p orbitals (Figure 8D). This result indicated that the light absorption was caused by the electronic transition from the N 2p to C 2p states (Zhao et al., 2018). The composite catalyst g-C_3_N_4_/γ-FeOOH was an indirect band gap (Figure 8B); the VBM was located at point Z, and the CBM was located between Point Q and Z instead of the high symmetry point. The energy bandwidth was significantly narrowed to 1.82 eV. The composition of the VBM and CBM of g-C_3_N_4_/γ-FeOOH primarily arose from the contribution of γ-FeOOH. The VBM of γ-FeOOH was mainly composed of O 2p and Fe 3d orbitals; in contrast, CBM was mainly composed of Fe 3d and only a small part of O 2p orbitals. The g-C_3_N_4_/γ-FeOOH structure did not introduce a local state in the forbidden band, primarily because the C−N bond on the surface of g-C_3_N_4_ and the Fe−O bond on the surface of γ-FeOOH were in a saturated coordination state. The bandgap of γ-FeOOH was 2.06 eV, and the bandgap was obviously narrowed after silver deposition (Figures 8F,G). There were spin-up impurity bands in the forbidden bands of Ag/γ-FeOOH and g-C_3_N_4_/Ag/γ-FeOOH, which may be attributed to the Ag 5s energy level (He et al., 2017b). The band structure of g-C_3_N_4_/Ag/γ-FeOOH became smoother, with an indirect band gap of 1.05 eV (Figure 6A). The further narrowing of the band gap increases the light absorption intensity of the catalyst and expands the absorption edge (Song et al., 2018); consequently, the local resonance at the interface significantly increases the strength of the electromagnetic field near the surface of Ag, thereby improving the separation efficiency of the e^−^-h^+^ pairs (Min et al., 2016; He et al., 2017b), which is also conducive to the generation of more photogenerated e^−^-h^+^ pairs in the visible region (Hou and Cronin, 2013; Zhao et al., 2015).

The above analysis revealed that the energy bandwidth of the catalysts gradually narrowed. Combined with the photocatalytic degradation experiment indicated that the composite catalyst could promote the absorption of visible light. The catalysts were all indirect bandgap semiconductors. Compared with direct bandgap semiconductors, the e^−^-h^+^ pairs of the indirect band gaps need momentum for recombination, which may inhibit the recombination of photo-generated e^−^-h^+^ pairs to a certain extent and enhance the photocatalytic efficiency.

### Product Analysis and Photodegradation Process

**FIGURE 9:**
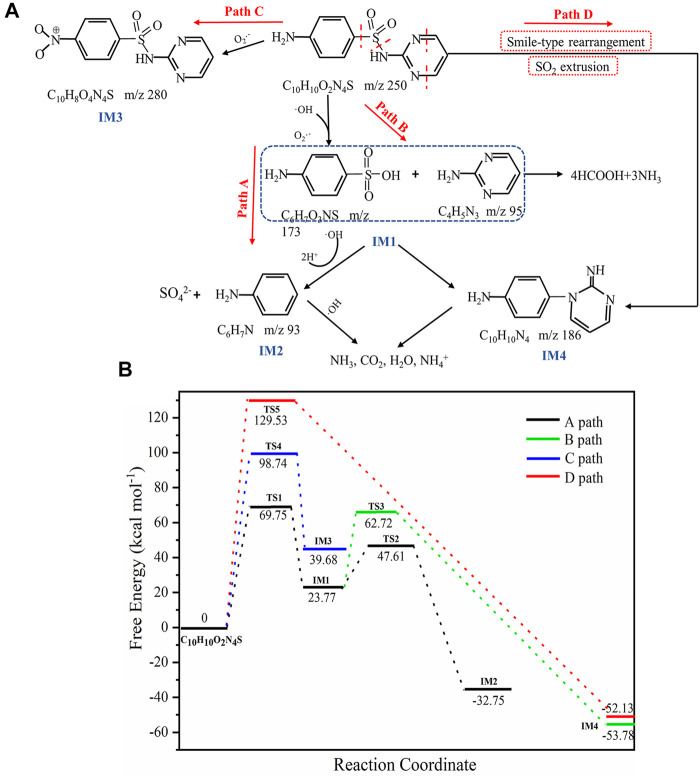
**(A)** Possible pathways for catalytic photodissociation of SDZ, **(B)** the potential energy profile, and the resulting transition state of the reaction.

Although previous studies have identified the byproducts of SDZ degradation, the intermediate products of degradation under different conditions were different. LC-MS was used to detect the intermediate product of SDZ catalyst degradation (Supplementary Table S4 and Supplementary Figures S4A–C). Combined with DFT calculations, four possible degradation pathways of SDZ were speculated (Figure 9A). The potential energy profile of the reaction is shown in Figure 9B. The optimized SDZ structure, the main intermediates of photocatalytic degradation, and the transition state are shown in Supplementary Figures S5A–B. According to previous studies, the potential photocatalysis cleavage sites of sulfa drugs are as follows (Boreen et al., 2004):

In path A, the S-N bond of SDZ was broken because of the attack of OH and O_2_^−^·, resulting in the production of IM1 (m/z = 173, sulfanilic acid, m/z = 95, 2-amino pyrimidine) (Figure 9A). The reaction needed to overcome the barrier of 69.75 kcal mol^−1^ while absorbing 23.77 kcal mol^−1^ of energy, which was corroborated by previous studies for the cleavage of the δ bond (Boreen et al., 2004). SO_2_ extrusion is an important site of further degradation of SAs by OH radicals (Wang et al., 2018). The product IM2 (m/z = 93, aniline) was produced by sulfanilic acid extruding SO_2_ under the action of OH. The reaction needed to overcome the reaction barrier of 23.84 kcal mol^−1^ while releasing 56.52 kcal mol^−1^ of energy. According to the Arrhenius equation, lower reaction energy barriers correspond to lower activation energy and greater rate constants of the chemical reaction (Zha et al., 2014). Therefore, the reaction rates of IM1 to IM2 were higher than that of the reaction producing IM1. In addition, the reaction heat was higher, indicating that the product IM2 can be generated stably, which also confirmed the breakage of the γ bond. The same reaction occurred in path D. First, the electrons of SDZ were transferred to the pores of the photocatalyst, leading to the formation of aniline radical cations, and then the product IM4 (m/z = 186, 4-[2-iminopyrimidine-1(2H)-yl]anline) was formed through Smile rearrangement and SO_2_ extrusion under the action of free radicals, which needed to overcome the reaction barrier of 129.53 kcal mol^−1^ and release 52.13 kcal mol^−1^ of energy. Because this reaction had a high reaction barrier, the reaction rate was slow. The intermediate product IM1 in path B produced IM4 through polymerization and desulfurization reactions (Wang et al., 2010). The product IM3 (m/z = 280, benzenesulfonamide) in path C was produced by direct oxidation of the N atom on SDZ by O_2_^−^ (Yang et al., 2018).

## Conclusion

In this study, the composite catalyst g-C_3_N_4_/Ag/γ-FeOOH was successfully prepared. Different photocatalysts had different catalytic effects, and the combination of the three had the highest degradation effect on the photocatalysis of SDZ. g-C_3_N_4_ can provide electrons for γ-FeOOH to convert Fe^3+^ into Fe^2+^, and γ-FeOOH can assist g-C_3_N_4_ by enhancing the absorption of visible light. The Ag ions were photo-deposited on the γ-FeOOH and g-C_3_N_4_ layers to separate e^−^-h^+^ pairs. The removal effect of SDZ was closely related to the loading of g-C_3_N_4_, the amount of catalyst, and other parameters. The composite catalyst g-C_3_N_4_/Ag/γ-FeOOH had a superior degradation effect on SDZ than g-C_3_N_4_ and γ-FeOOH. When the loading of g-C_3_N_4_ was 2.5%, the degradation effect of SDZ was higher than 7.5%. The degradation promotion rate of g-C_3_N_4_ (2.5 wt%)/Ag/γ-FeOOH for SDZ reached 135%, whereas that of g-C_3_N_4_ (7.5 wt%)/Ag/γ-FeOOH was 57.5%. 2 mg/L g-C_3_N_4_(5.0 wt%)/Ag/γ-FeOOH had the strongest effect and promoted the degradation of SDZ at a rate of 258.75% within 600 min. Free radical capture experiments revealed that OH was the main free radical during SDZ degradation. DFT calculations showed that the enhancement of photocatalytic activity was related to the narrowing of the forbidden band of the catalyst structure and the local electron density of the VB. The band gap of the composite catalyst was gradually reduced from 2.7 to 1.05 eV, and the electrons generated local resonance, which can increase the absorption of photons by the catalyst and promote the migration and separation of surface photogenerated carriers. The degradation path was inferred according to LC-MS and DFT calculations. The analysis confirmed that the degradation path of SDZ primarily included Smiles-type rearrangement, SO_2_ extrusion, and S-N bond cleavage processes. This work not only provides a method for improving the catalytic performance of novel metal-free g-C_3_N_4_-based semiconductor catalysts but also has an important theoretical significance for understanding the efficient removal mechanism of catalysts for pollutants, which also provides a new idea for the removal of emerging pollutants by photocatalysis.

## Data Availability

The original contributions presented in the study are included in the article/Supplementary Material, further inquiries can be directed to the corresponding authors.
