# Supplementary figures and images for: Combined the Photocatalysis and Fenton-like Reaction to Efficiently Remove Sulfadiazine in Water Using g-C3N4/Ag/γ-FeOOH: Insights Into the Degradation Pathway From Density Functional Theory
Source: Front Chem. 2021 Oct 5;9:742459. doi: 10.3389/fchem.2021.742459 (PMC8525599; doi:10.3389/fchem.2021.742459)

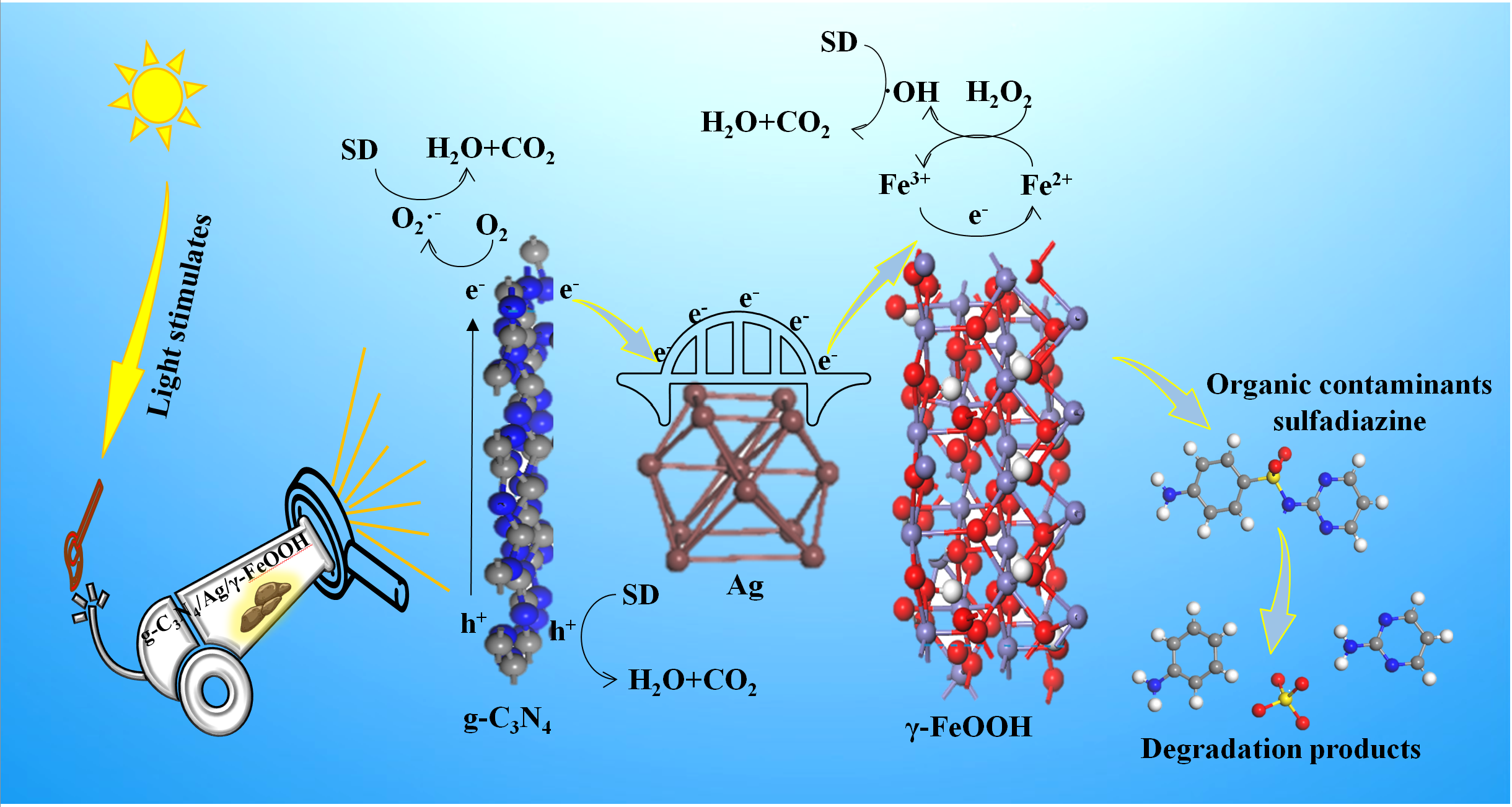

Supplement: Supplementary file 1 [file Image1.TIF]
