# Supplementary material for: Combined the Photocatalysis and Fenton-like Reaction to Efficiently Remove Sulfadiazine in Water Using g-C3N4/Ag/γ-FeOOH: Insights Into the Degradation Pathway From Density Functional Theory
Source: Front Chem. 2021 Oct 5;9:742459. doi: 10.3389/fchem.2021.742459 (PMC8525599; doi:10.3389/fchem.2021.742459)
Supplement: Supplementary file 2 [file DataSheet1.docx]

**^[[1]](#footnote-1)^Appendix−Supporting information for:**

**Combined the photocatalysis and Fenton-like reaction to efficiently remove sulfadiazine in water using g-C_3_N_4_/Ag/γ-FeOOH: insights into the degradation pathway from density functional theory**

**Summary: this file includes 7 pages, 1 texts, 5 figures, 7 tables on:**

◆ The details of g-C_3_N_4_/Ag/γ-FeOOH synthesis methods **(Text S1).**

- Image of the device that simulates the degradation of SDZ by visible light (**Figure S1**)

◆ The surface-scanned electron image of g-C_3_N_4_(5wt%)/Ag/γ-FeOOH (**Figure S2**)

◆ The degradation kinetics of g-C_3_N_4_ for SDZ degradation **(Table S1).**

◆ The degradation kinetics of γ-FeOOH for SDZ degradation (**Table S2**)

◆ The degradation kinetics of g-C_3_N_4_(2.5wt%,5.0wt%7.5wt%)/Ag/γ-FeOOH for SDZ degradation (**Table S3**)

◆ The optimized single-layer planar structure of g-C_3_N_4_ (**Figure S3a**); The optimized lattice structure of γ-FeOOH, g-C_3_N_4_/γ-FeOOH, g-C_3_N_4_/Ag/γ-FeOOH (**Figure S3b, c, d**).

◆ SDZ and SDZ catalytic photolysis intermediates (**Table S4**)

◆ Mass spectrogram of 75% catalytic degradation of SDZ by g-C_3_N_4_(5.0wt%)/ Ag/γ-FeOOH (**Figure S4a**); Photodissociation mass spectrometry of SDZ with isopropanol (**Figure S4b**); Photodissociation mass spectrometry of SDZ with sorbic acid (**Figure S4c**).

◆ The main intermediates of SDZ photocatalytic degradation (**Figure S5a**); The transition state of SDZ photocatalytic degradation (**Figure S5b**).

- Cartesian Coordinates of g-C_3_N_4_ (**Table S5**).
- Cartesian Coordinates of γ-FeOOH (**Table S6**).
- Cartesian Coordinates of g-C_3_N_4_/Ag/γ-FeOOH (**Table S7**).

**Text S1: The details of g-C_3_N_4_/Ag/γ-FeOOH synthesis methods:** 0.4 g γ-FeOOH was dispersed in 400 mL of deionized water under sonication for 30 min. The silver was deposited on the surface of γ-FeOOH with a photodeposition method: 10 mL of AgNO_3_ solution (1.26 mg/mL) was poured into the γ-FeOOH solution and then was irradiated under Xe lamp for 60 min. Then 1 mg/mL g-C_3_N_4_ sheets dispersion(10, 20, and 40 mL) was poured into the solution and maintained the temperature at 75◦C for 1 h to get g-C_3_N_4_/Ag/γ-FeOOH with the loading of 2.5, 5.0, and 7.5wt% g-C_3_N_4_. Then the solution was filtrated, washed with absolute ethyl alcohol and deionized water, and dried at 60◦C for 12 h to get g-C_3_N_4_/Ag/γ-FeOOH.


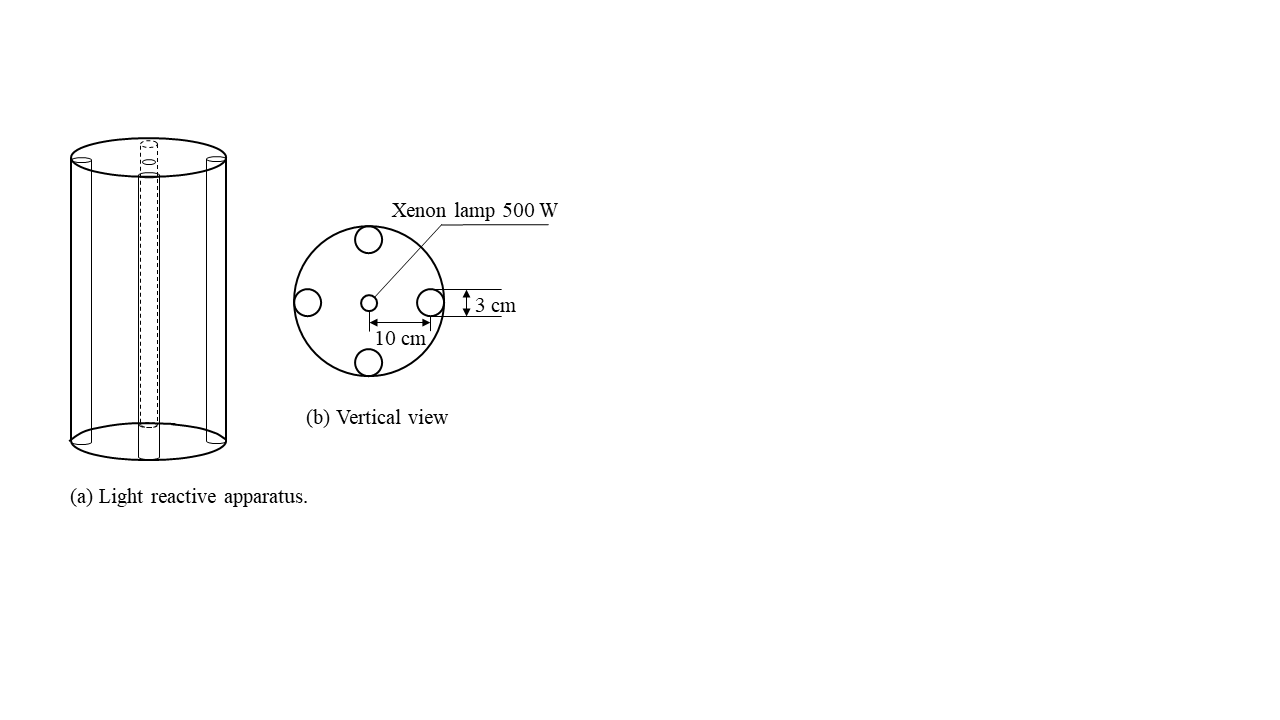


**Figure S1.** Diagram of the device that simulates the degradation of SDZ by visible light.

**
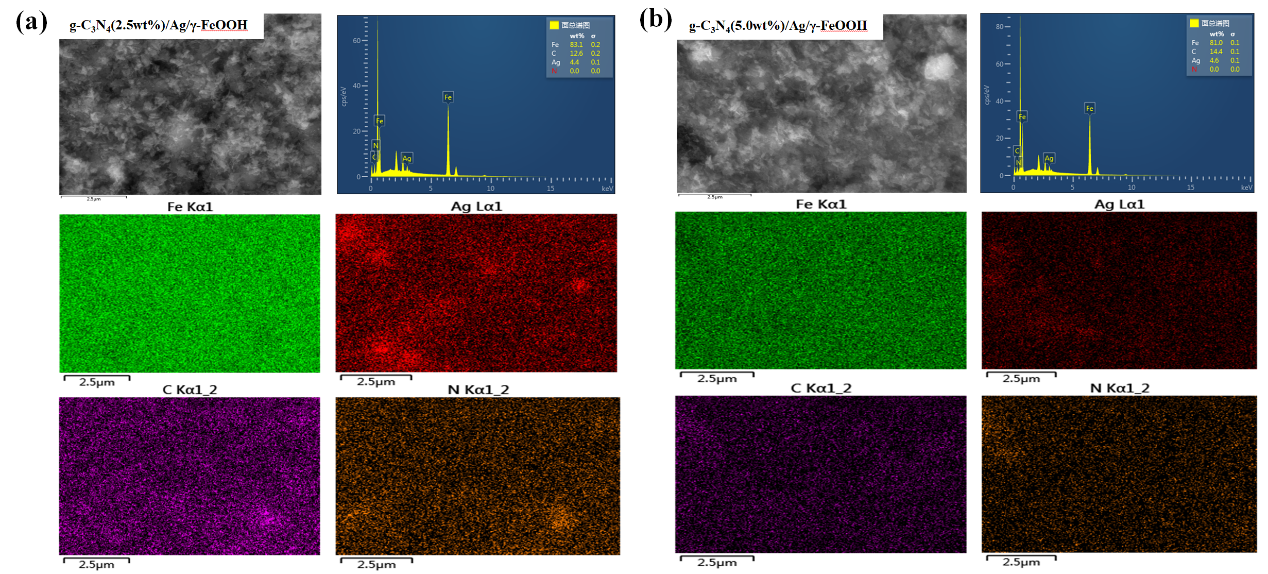
**

**Figure S2.** The surface-scanned electron image of g-C_3_N_4_(2.5 wt%, 5wt%)/Ag/γ-FeOOH

**Table S1.** The kinetic equations and parameters of g-C_3_N_4_ for SDZ degradation

| Concentration（mg/L） | Speed constant（10^-3^min^-1^） | Half-life period（h） | Correlation coefficient R^2^ | Promoting ratio ζ |
| --- | --- | --- | --- | --- |
| 0 | 0.80 | 14.44 | 0.98 | / |
| 5 | 0.83 | 13.92 | 0.99 | 3.75% |
| 10 | 1.39 | 8.31 | 0.99 | 73.75% |
| 15 | 1.29 | 8.96 | 0.99 | 61.25% |

**Table S2.** The kinetic equations and parameters of γ-FeOOH for SDZ degradation

| Concentration（mg/L） | Speed constant （10^-3^min^-1^） | Half-life period（h） | Correlation coefficient R^2^ | Promoting ratio ζ |
| --- | --- | --- | --- | --- |
| 5 | 0.9 | 12.84 | 0.99 | 12.5% |
| 10 | 1.03 | 11.22 | 0.98 | 28.8% |
| 25 | 1.37 | 8.43 | 0.96 | 71.3% |
| 50 | 1.42 | 8.14 | 0.99 | 77.5% |

**Table S3.** The kinetic equations and parameters of g-C_3_N_4_/Ag/γ-FeOOH for SDZ degradation

| Catalysts | Concentration（mg/L） | | | Speed constant （10^-3^min^-1^） | | Half-life period（h） | | Correlation coefficient R^2^ | | Promoting ratio ζ |
| --- | --- | --- | --- | --- | --- | --- | --- | --- | --- | --- |
| g-C_3_N_4_(2.5wt%)/Ag/  γ-FeOOH | | 1 | 1.88 | | 6.14 | | 0.98 | | 135.0% | |
|  |  | 2 | 1.93 | | 5.99 | | 0.99 | | 141.3% | |
|  |  | 5 | 1.8 | | 6.42 | | 0.97 | | 125.0% | |
|  |  | 10 | 1.72 | | 6.72 | | 0.97 | | 115.0% | |
|  |  | 25 | 1.28 | | 9.03 | | 0.97 | | 60.0% | |
|  |  | 50 | 1.66 | | 6.96 | | 0.97 | | 107.5% | |
| g-C_3_N_4_(5.0wt%)/Ag/  γ-FeOOH | | 1 | | 1.91 | 6.05 | | 0.95 | | 138.8% | |
|  |  | 2 | | 2.87 | 4.03 | | 0.97 | | 258.8% | |
|  |  | 5 | | 1.41 | 8.19 | | 0.93 | | 76.3% | |
|  |  | 10 | | 2.27 | 5.09 | | 0.97 | | 183.8% | |
|  |  | 25 | | 1.48 | 7.81 | | 0.94 | | 85.0% | |
|  |  | 50 | | 1.11 | 10.41 | | 0.93 | | 38.8% | |
| g-C_3_N_4_(7.5wt%)/Ag/  γ-FeOOH | | 1 | | 1.26 | 9.17 | | 0.99 | | 57.5% | |
|  |  | 2 | | 1.54 | 7.50 | | 0.99 | | 92.5% | |
|  |  | 5 | | 1.56 | 7.41 | | 0.99 | | 95.0% | |
|  |  | 10 | | 1.48 | 7.81 | | 0.99 | | 85.0% | |
|  |  | 25 | | 1.41 | 8.19 | | 0.99 | | 76.3% | |
|  |  | 50 | | 2.45 | 4.72 | | 0.99 | | 206.3% | |

**
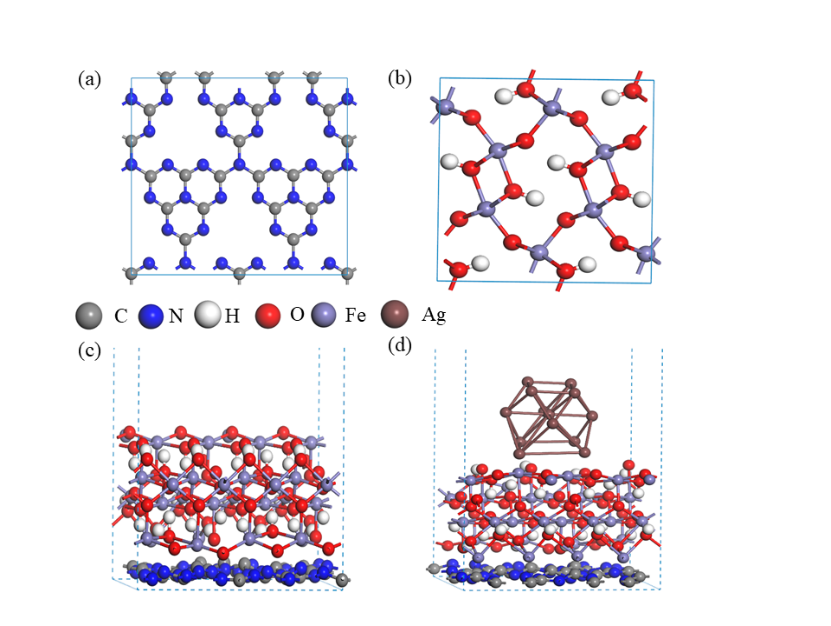
**

**Figure S3.** (a) The optimized single-layer planar structure of g-C_3_N_4_; (b,c,d) The optimized lattice structure of γ-FeOOH, g-C_3_N_4_/γ-FeOOH, g-C_3_N_4_/Ag/γ-FeOOH.

**Table S4.** SDZ and SDZ catalytic photolysis intermediates

| [molecular](javascript:;) [formula](javascript:;) | g-C_3_N_4_(5.0wt%)/Ag/γ-FeOOH catalysis | | | | | |
| --- | --- | --- | --- | --- | --- | --- |
|  | SD was degraded by 25% | SD was degraded by 75% | Add isopropyl alcohol | | Add sorbic acid |  |
| C_10_H_10_O_2_N_4_S | **√** | **√** | **√** | **√** | | |
| C_6_H_7_N | **√** | **√** |  | **√** | | |
| C_6_H_7_O_3_NS | **√** |  | **√** |  | | |
| C_10_H_8_O_4_N_4_S |  | **√** | **√** |  | | |
| C_10_H_10_N_4_ | **√** | **√** | **√** | **√** | | |


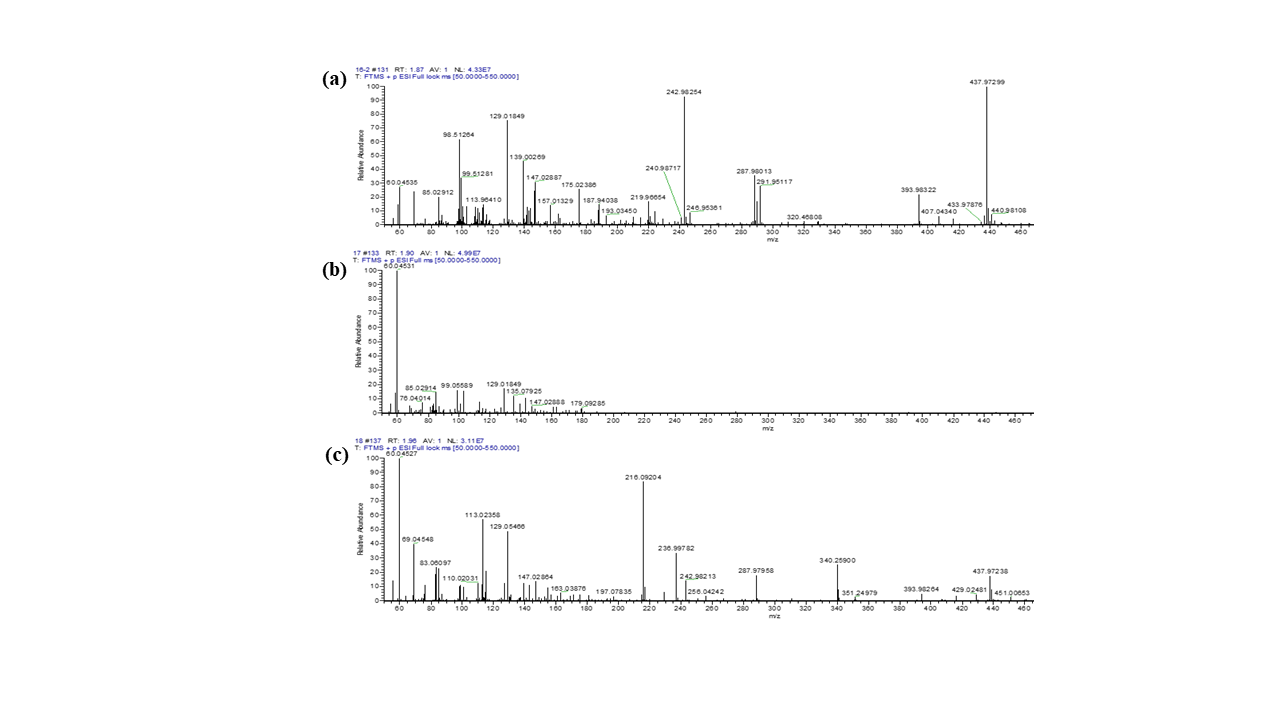


**Figure S4.** (a) Mass spectrogram of 75% catalytic degradation of SDZ by g-C_3_N_4_(5.0wt%)/Ag/γ-FeOOH; (b) Photodissociation mass spectrometry of SDZ with isopropanol; (c) Photodissociation mass spectrometry of SDZ with sorbic acid


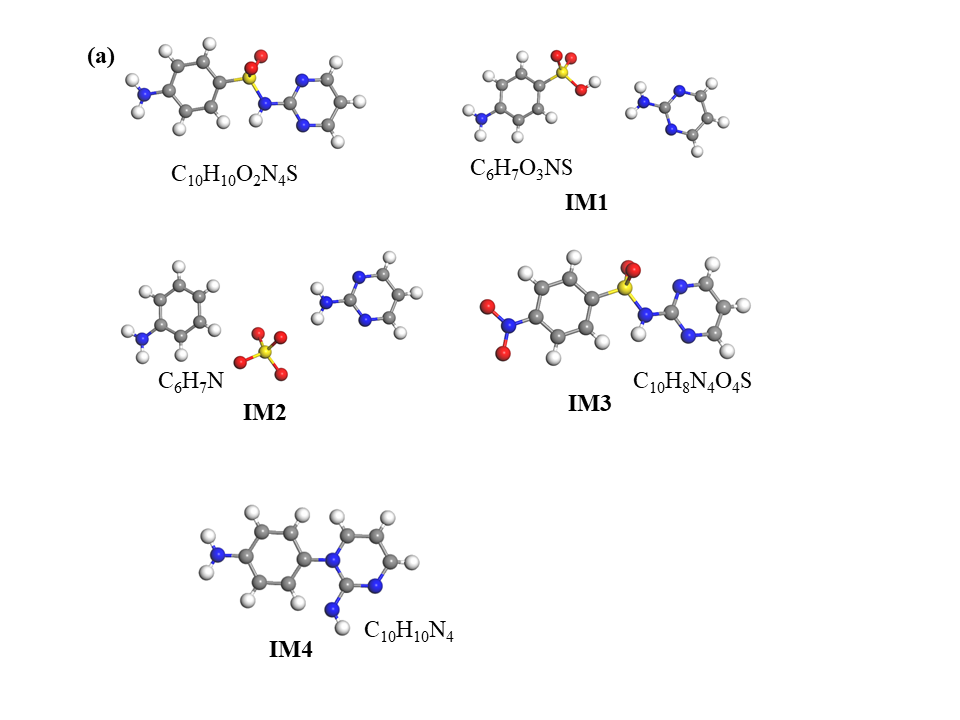


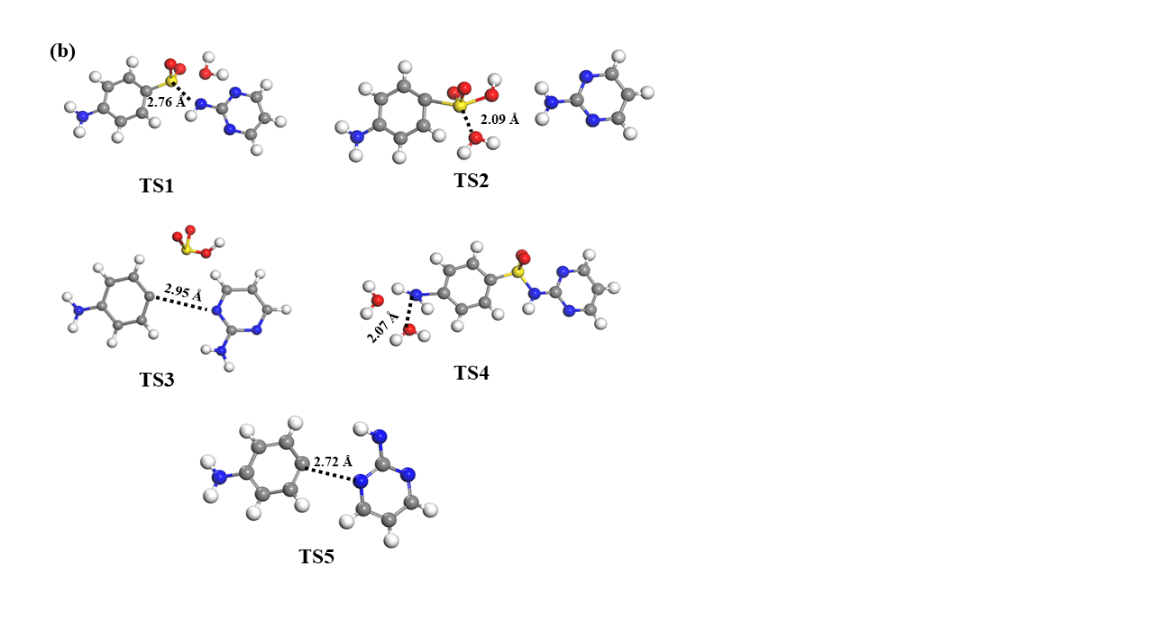


**Figure S5.** (a)The main intermediates of SDZ photocatalytic degradation; (b) The transition state of SDZ photocatalytic degradation.

**Table S5.** Cartesian Coordinates of g-C_3_N_4_

| Atoms | X | Y | Z |
| --- | --- | --- | --- |
| C | -2.29100000 | 12.35280000 | 1.78440000 |
| C | -1.20370000 | 10.33590000 | 1.78440000 |
| C | 0.00000000 | 12.42080000 | 1.78440000 |
| C | 0.00000000 | 8.39190000 | 1.78440000 |
| C | 1.20370000 | 10.33590000 | 1.78440000 |
| C | 2.29100000 | 12.35280000 | 1.78440000 |
| C | 1.27640000 | 6.17610000 | 1.78440000 |
| C | 2.36380000 | 4.15910000 | 1.78440000 |
| C | 3.56750000 | 6.24400000 | 1.78440000 |
| C | 3.56750000 | 2.21520000 | 1.78440000 |
| C | 4.77120000 | 4.15910000 | 1.78440000 |
| C | 5.85850000 | 6.17610000 | 1.78440000 |
| N | 0.00000000 | 6.91500000 | 1.78440000 |
| N | -1.16260000 | 13.07030000 | 1.78440000 |
| N | -2.34660000 | 11.02210000 | 1.78440000 |
| N | 0.00000000 | 11.03090000 | 1.78440000 |
| N | -1.18200000 | 9.00770000 | 1.78440000 |
| N | 1.18200000 | 9.00770000 | 1.78440000 |
| N | 2.34660000 | 11.02210000 | 1.78440000 |
| N | 1.16260000 | 13.07030000 | 1.78440000 |
| N | 3.56750000 | 0.73830000 | 1.78440000 |
| N | 2.40490000 | 6.89360000 | 1.78440000 |
| N | 1.22090000 | 4.84540000 | 1.78440000 |
| N | 3.56750000 | 4.85420000 | 1.78440000 |
| N | 2.38550000 | 2.83100000 | 1.78440000 |
| N | 4.74950000 | 2.83100000 | 1.78440000 |
| N | 5.91410000 | 4.84540000 | 1.78440000 |
| N | 4.73010000 | 6.89360000 | 1.78440000 |
| C | 4.84390000 | -0.00060000 | 1.78440000 |
| C | 2.29100000 | -0.00060000 | 1.78440000 |
| C | -1.27640000 | 6.17610000 | 1.78440000 |
| N | 7.13500000 | 6.91500000 | 1.78440000 |
| N | -3.56750000 | 13.09170000 | 1.78440000 |
| N | 3.56750000 | 13.09170000 | 1.78440000 |
| C | 2.29100000 | 0.00060000 | 5.35320000 |
| C | 1.20370000 | 2.01760000 | 5.35320000 |
| C | 0.00000000 | -0.06730000 | 5.35320000 |
| C | 0.00000000 | 3.96150000 | 5.35320000 |
| C | -1.20370000 | 2.01760000 | 5.35320000 |
| C | -2.29100000 | 0.00060000 | 5.35320000 |
| C | 5.85850000 | 6.17730000 | 5.35320000 |
| C | 4.77120000 | 8.19430000 | 5.35320000 |
| C | 3.56750000 | 6.10940000 | 5.35320000 |
| C | 3.56750000 | 10.13820000 | 5.35320000 |
| C | 2.36380000 | 8.19430000 | 5.35320000 |
| C | 1.27640000 | 6.17730000 | 5.35320000 |
| N | 0.00000000 | 5.43840000 | 5.35320000 |
| N | 1.16260000 | -0.71690000 | 5.35320000 |
| N | 2.34660000 | 1.33130000 | 5.35320000 |
| N | 0.00000000 | 1.32250000 | 5.35320000 |
| N | 1.18200000 | 3.34570000 | 5.35320000 |
| N | -1.18200000 | 3.34570000 | 5.35320000 |
| N | -2.34660000 | 1.33130000 | 5.35320000 |
| N | -1.16260000 | -0.71690000 | 5.35320000 |
| N | 3.56750000 | 11.61510000 | 5.35320000 |
| N | 4.73010000 | 5.45980000 | 5.35320000 |
| N | 5.91410000 | 7.50800000 | 5.35320000 |
| N | 3.56750000 | 7.49920000 | 5.35320000 |
| N | 4.74950000 | 9.52240000 | 5.35320000 |
| N | 2.38550000 | 9.52240000 | 5.35320000 |
| N | 1.22090000 | 7.50800000 | 5.35320000 |
| N | 2.40490000 | 5.45980000 | 5.35320000 |
| C | 2.29100000 | 12.35400000 | 5.35320000 |
| C | 4.84390000 | 12.35400000 | 5.35320000 |
| C | -1.27640000 | 6.17730000 | 5.35320000 |
| N | 7.13500000 | 5.43840000 | 5.35320000 |
| N | -3.56750000 | -0.73830000 | 5.35320000 |
| N | 3.56750000 | -0.73830000 | 5.35320000 |

**Table S6.** Cartesian Coordinates of γ-FeOOH

| Atoms | X | Y | Z |
| --- | --- | --- | --- |
| Fe | 0.76800000 | 2.56660000 | 6.49460000 |
| Fe | 0.76800000 | 4.42660000 | 1.45110000 |
| Fe | 2.30390000 | 2.09550000 | 3.59230000 |
| Fe | 2.30390000 | 0.23550000 | 8.63580000 |
| H | 0.76800000 | 4.20490000 | 4.15330000 |
| H | 0.76800000 | 2.78820000 | 9.19680000 |
| H | 2.30390000 | 0.45720000 | 5.93360000 |
| O | 0.76800000 | 0.91960000 | 3.08870000 |
| O | 0.76800000 | 1.41150000 | 8.13210000 |
| O | 2.30390000 | 3.74250000 | 6.99830000 |
| O | 2.30390000 | 3.25060000 | 1.95480000 |
| O | 0.76800000 | 3.25700000 | 4.48960000 |
| O | 0.76800000 | 3.73610000 | 9.53310000 |
| O | 2.30390000 | 1.40510000 | 5.59720000 |
| Fe | 3.83980000 | 2.56660000 | 6.49460000 |
| Fe | 3.83980000 | 4.42660000 | 1.45110000 |
| Fe | 5.37570000 | 2.09550000 | 3.59230000 |
| Fe | 5.37570000 | 0.23550000 | 8.63580000 |
| H | 3.83980000 | 4.20490000 | 4.15330000 |
| H | 3.83980000 | 2.78820000 | 9.19680000 |
| H | 5.37570000 | 0.45720000 | 5.93360000 |
| O | 3.83980000 | 0.91960000 | 3.08870000 |
| O | 3.83980000 | 1.41150000 | 8.13210000 |
| O | 5.37570000 | 3.74250000 | 6.99830000 |
| O | 5.37570000 | 3.25060000 | 1.95480000 |
| O | 3.83980000 | 3.25700000 | 4.48960000 |
| O | 3.83980000 | 3.73610000 | 9.53310000 |
| O | 5.37570000 | 1.40510000 | 5.59720000 |
| Fe | 0.76800000 | 7.22870000 | 6.49460000 |
| Fe | 0.76800000 | 9.08870000 | 1.45110000 |
| Fe | 2.30390000 | 6.75760000 | 3.59230000 |
| Fe | 2.30390000 | 4.89760000 | 8.63580000 |
| H | 0.76800000 | 8.86700000 | 4.15330000 |
| H | 2.30390000 | 5.11930000 | 5.93360000 |
| H | 2.30390000 | 6.53600000 | 0.89020000 |
| O | 0.76800000 | 5.58170000 | 3.08870000 |
| O | 0.76800000 | 6.07360000 | 8.13210000 |
| O | 2.30390000 | 8.40460000 | 6.99830000 |
| O | 2.30390000 | 7.91270000 | 1.95480000 |
| O | 0.76800000 | 7.91910000 | 4.48960000 |
| O | 2.30390000 | 6.06720000 | 5.59720000 |
| O | 2.30390000 | 5.58810000 | 0.55380000 |
| Fe | 3.83980000 | 7.22870000 | 6.49460000 |
| Fe | 3.83980000 | 9.08870000 | 1.45110000 |
| Fe | 5.37570000 | 6.75760000 | 3.59230000 |
| Fe | 5.37570000 | 4.89760000 | 8.63580000 |
| H | 3.83980000 | 8.86700000 | 4.15330000 |
| H | 5.37570000 | 5.11930000 | 5.93360000 |
| H | 5.37570000 | 6.53600000 | 0.89020000 |
| O | 3.83980000 | 5.58170000 | 3.08870000 |
| O | 3.83980000 | 6.07360000 | 8.13210000 |
| O | 5.37570000 | 8.40460000 | 6.99830000 |
| O | 5.37570000 | 7.91270000 | 1.95480000 |
| O | 3.83980000 | 7.91910000 | 4.48960000 |
| O | 5.37570000 | 6.06720000 | 5.59720000 |
| O | 5.37570000 | 5.58810000 | 0.55380000 |
| H | 2.30390000 | 1.87390000 | 10.97710000 |
| O | 2.30390000 | 0.92600000 | 10.64070000 |
| H | 5.37570000 | 1.87390000 | 10.97710000 |
| O | 5.37570000 | 0.92600000 | 10.64070000 |
| H | 0.76800000 | -1.87390000 | 9.19680000 |
| O | 0.76800000 | -0.92600000 | 9.53310000 |
| H | 3.83980000 | -1.87390000 | 9.19680000 |
| O | 3.83980000 | -0.92600000 | 9.53310000 |
| Fe | 6.91160000 | 2.56660000 | 6.49460000 |
| Fe | 0.76800000 | 4.42660000 | 11.53800000 |
| Fe | 6.91160000 | 4.42660000 | 1.45110000 |
| Fe | 8.44740000 | 2.09550000 | 3.59230000 |
| Fe | 2.30390000 | 9.55970000 | 8.63580000 |
| Fe | 8.44740000 | 0.23550000 | 8.63580000 |
| O | 0.76800000 | 10.24380000 | 3.08870000 |
| O | 6.91160000 | 0.91960000 | 3.08870000 |
| O | 6.91160000 | 1.41150000 | 8.13210000 |
| O | 6.91160000 | 3.25700000 | 4.48960000 |
| O | 0.76800000 | 3.73610000 | -0.55380000 |
| O | 6.91160000 | 3.73610000 | 9.53310000 |
| Fe | -2.30380000 | 2.56660000 | 6.49460000 |
| Fe | -2.30380000 | 4.42660000 | 1.45110000 |
| Fe | 3.83980000 | 4.42660000 | 11.53800000 |
| Fe | -0.76790000 | 2.09550000 | 3.59230000 |
| Fe | -0.76790000 | 0.23550000 | 8.63580000 |
| Fe | 5.37570000 | 9.55970000 | 8.63580000 |
| O | 3.83980000 | 10.24380000 | 3.08870000 |
| O | -0.76790000 | 3.74250000 | 6.99830000 |
| O | -0.76790000 | 3.25060000 | 1.95480000 |
| O | 3.83980000 | 3.73610000 | -0.55380000 |
| O | -0.76790000 | 1.40510000 | 5.59720000 |
| Fe | 6.91160000 | 7.22870000 | 6.49460000 |
| Fe | 0.76800000 | -0.23550000 | 1.45110000 |
| Fe | 0.76800000 | -0.23550000 | 11.53800000 |
| Fe | 6.91160000 | -0.23550000 | 11.53800000 |
| Fe | 6.91160000 | 9.08870000 | 1.45110000 |
| Fe | 8.44740000 | 6.75760000 | 3.59230000 |
| Fe | 2.30390000 | 4.89760000 | -1.45110000 |
| Fe | 8.44740000 | 4.89760000 | 8.63580000 |
| O | 6.91160000 | 5.58170000 | 3.08870000 |
| O | 6.91160000 | 6.07360000 | 8.13210000 |
| O | 2.30390000 | -0.91960000 | 6.99830000 |
| O | 6.91160000 | 7.91910000 | 4.48960000 |
| O | 2.30390000 | 5.58810000 | 10.64070000 |
| Fe | -2.30380000 | 7.22870000 | 6.49460000 |
| Fe | -2.30380000 | 9.08870000 | 1.45110000 |
| Fe | 3.83980000 | -0.23550000 | 1.45110000 |
| Fe | 3.83980000 | -0.23550000 | 11.53800000 |
| Fe | -0.76790000 | 6.75760000 | 3.59230000 |
| Fe | -0.76790000 | 4.89760000 | 8.63580000 |
| Fe | 5.37570000 | 4.89760000 | -1.45110000 |
| O | -0.76790000 | 8.40460000 | 6.99830000 |
| O | 5.37570000 | -0.91960000 | 6.99830000 |
| O | -0.76790000 | 7.91270000 | 1.95480000 |
| O | -0.76790000 | 6.06720000 | 5.59720000 |
| O | -0.76790000 | 5.58810000 | 0.55380000 |
| O | 5.37570000 | 5.58810000 | 10.64070000 |
| O | 2.30390000 | 10.25020000 | 0.55380000 |
| O | -0.76790000 | 10.25020000 | 0.55380000 |
| O | 5.37570000 | 10.25020000 | 0.55380000 |
| O | 0.76800000 | 8.39820000 | -0.55380000 |
| O | 6.91160000 | -0.92600000 | 9.53310000 |
| O | 3.83980000 | 8.39820000 | -0.55380000 |

**Table S7.** Cartesian Coordinates of g-C_3_N_4_/Ag/γ-FeOOH

| Atoms | X | Y | Z |
| --- | --- | --- | --- |
| O | 5.77210000 | 12.89180000 | 6.74450000 |
| Fe | 0.79260000 | 10.00940000 | 4.83330000 |
| Fe | 0.79260000 | 1.68060000 | 2.82820000 |
| Fe | 2.31710000 | 11.76530000 | 2.26480000 |
| Fe | 2.45240000 | 7.01350000 | 4.58160000 |
| H | 0.79260000 | 12.69820000 | 4.45710000 |
| H | 0.79260000 | 8.26790000 | 6.77900000 |
| H | 2.45240000 | 9.04080000 | 2.95280000 |
| H | 2.45240000 | 6.43690000 | 7.30230000 |
| O | 0.90840000 | 4.82240000 | 8.60040000 |
| O | 0.79260000 | 8.12280000 | 5.08980000 |
| O | 2.45240000 | 10.41620000 | 6.00790000 |
| O | 2.45240000 | 0.57120000 | 2.32000000 |
| O | 0.77430000 | 11.60500000 | 3.93800000 |
| O | 0.79260000 | 8.64430000 | 7.67980000 |
| O | 2.42730000 | 10.06400000 | 2.98410000 |
| O | 2.45240000 | 6.06040000 | 6.40180000 |
| Fe | 4.11220000 | 10.00940000 | 4.83330000 |
| Fe | 4.03830000 | 1.66220000 | 2.48530000 |
| Fe | 5.40570000 | 11.68130000 | 2.40830000 |
| Fe | 5.77210000 | 7.01350000 | 4.58160000 |
| H | 4.11220000 | 12.69820000 | 4.45710000 |
| H | 4.11220000 | 8.26790000 | 6.77900000 |
| H | 5.77210000 | 9.04080000 | 2.95280000 |
| O | 3.73610000 | 4.08420000 | 7.95060000 |
| O | 4.11220000 | 8.12280000 | 5.08980000 |
| O | 5.77210000 | 10.41620000 | 6.00790000 |
| O | 5.60010000 | 13.60060000 | 2.05070000 |
| O | 4.11220000 | 11.85260000 | 4.00110000 |
| O | 4.11220000 | 8.64430000 | 7.67980000 |
| O | 5.76080000 | 10.17710000 | 3.32250000 |
| O | 5.77210000 | 6.06040000 | 6.40180000 |
| Fe | 7.43190000 | 13.01480000 | 8.16900000 |
| Fe | 7.43190000 | 4.68590000 | 6.16400000 |
| Fe | 8.58630000 | 1.58470000 | 6.75450000 |
| Fe | 9.15350000 | 9.89570000 | 8.45240000 |
| H | 7.43190000 | 5.26240000 | 3.44320000 |
| H | 9.09170000 | 3.43160000 | 3.96660000 |
| O | 7.43190000 | 1.28320000 | 4.73790000 |
| O | 7.43190000 | 11.12810000 | 8.42560000 |
| O | 9.09170000 | 3.57670000 | 5.65580000 |
| O | 7.43190000 | 1.81300000 | 7.33690000 |
| O | 7.43190000 | 5.63900000 | 4.34400000 |
| O | 9.09150000 | 3.06540000 | 3.18800000 |
| Fe | 10.75150000 | 4.68590000 | 6.16400000 |
| Fe | 12.41140000 | 1.69000000 | 5.91230000 |
| Fe | 12.41140000 | 10.01890000 | 7.91740000 |
| H | 10.86240000 | 2.83900000 | 8.15320000 |
| H | 10.75150000 | 5.26240000 | 3.44320000 |
| O | 10.75150000 | 1.28320000 | 4.73790000 |
| O | 10.75150000 | 11.12810000 | 8.42560000 |
| O | 12.41110000 | 7.28860000 | 2.68190000 |
| O | 12.41140000 | 3.57670000 | 5.65580000 |
| O | 10.75150000 | 1.81300000 | 7.33690000 |
| O | 10.75150000 | 5.63900000 | 4.34400000 |
| Fe | 7.43190000 | 10.00940000 | 4.83330000 |
| Fe | 7.43190000 | 1.68060000 | 2.82820000 |
| Fe | 8.67650000 | 11.67170000 | 2.35990000 |
| Fe | 9.09170000 | 7.01350000 | 4.58160000 |
| H | 7.43190000 | 12.69820000 | 4.45710000 |
| H | 7.43190000 | 8.26790000 | 6.77900000 |
| H | 9.09170000 | 9.04080000 | 2.95280000 |
| H | 9.09170000 | 6.43690000 | 7.30230000 |
| O | 7.46950000 | 4.64840000 | 8.35260000 |
| O | 7.43190000 | 8.12280000 | 5.08980000 |
| O | 9.09170000 | 10.41620000 | 6.00790000 |
| O | 9.11620000 | 13.61000000 | 2.19740000 |
| O | 7.43190000 | 11.85260000 | 4.00110000 |
| O | 7.25080000 | 8.37420000 | 7.86360000 |
| O | 9.09620000 | 9.71480000 | 3.41600000 |
| O | 9.09170000 | 6.06040000 | 6.40180000 |
| Fe | 10.75150000 | 10.00940000 | 4.83330000 |
| Fe | 10.75150000 | 1.68060000 | 2.82820000 |
| Fe | 12.09430000 | 11.67270000 | 2.31060000 |
| Fe | 12.41140000 | 7.01350000 | 4.58160000 |
| H | 10.75150000 | 12.69820000 | 4.45710000 |
| H | 10.75150000 | 8.26790000 | 6.77900000 |
| H | 12.41140000 | 9.04080000 | 2.95280000 |
| O | 10.84610000 | 5.06350000 | 8.36140000 |
| O | 10.75150000 | 8.12280000 | 5.08980000 |
| O | 12.41140000 | 10.41620000 | 6.00790000 |
| O | -0.86720000 | 13.61610000 | 2.32000000 |
| O | 10.75150000 | 11.85260000 | 4.00110000 |
| O | 10.74730000 | 8.50200000 | 7.84040000 |
| O | 12.38100000 | 10.18000000 | 3.41980000 |
| O | 12.41140000 | 6.06040000 | 6.40180000 |
| Ag | 6.57450000 | 6.41840000 | 12.53970000 |
| Ag | 9.45560000 | 6.25220000 | 12.67680000 |
| Ag | 8.21640000 | 4.54590000 | 10.33660000 |
| Ag | 3.69360000 | 6.58450000 | 12.40240000 |
| Ag | 5.15120000 | 8.26460000 | 14.24600000 |
| Ag | 6.76120000 | 9.26190000 | 12.06440000 |
| Ag | 4.37990000 | 7.91030000 | 9.80390000 |
| Ag | 4.96450000 | 5.42110000 | 14.72130000 |
| Ag | 6.41640000 | 3.37990000 | 12.49880000 |
| Ag | 4.58190000 | 4.78170000 | 10.38330000 |
| Ag | 7.84540000 | 5.25490000 | 14.85850000 |
| Ag | 8.03210000 | 8.09840000 | 14.38330000 |
| O | -0.86720000 | 12.89180000 | 6.74450000 |
| Ag | 8.76870000 | 7.79690000 | 9.70060000 |
| C | 4.47000000 | -0.00510000 | 0.56490000 |
| C | 2.09440000 | -0.00510000 | 0.56490000 |
| N | 13.17250000 | 7.34450000 | 0.98420000 |
| N | 13.19080000 | 11.61940000 | 0.87370000 |
| N | 1.04440000 | 0.75260000 | 0.56490000 |
| N | 3.25260000 | 13.81640000 | 0.74660000 |
| C | 11.10930000 | -0.00510000 | 0.56490000 |
| C | 11.10930000 | 13.03980000 | 0.56490000 |
| C | 12.08010000 | 10.83120000 | 0.81810000 |
| C | 8.73370000 | -0.00510000 | 0.56490000 |
| C | -1.25190000 | 6.51410000 | 0.72850000 |
| N | 11.05760000 | 11.63460000 | 0.56490000 |
| N | 7.65090000 | 0.76700000 | 0.76520000 |
| N | -3.35710000 | 13.82000000 | 0.56490000 |
| N | 9.92150000 | 13.82000000 | 0.56490000 |
| Fe | 0.79260000 | -0.03010000 | 8.16900000 |
| Fe | 14.07120000 | 4.68590000 | 6.16400000 |
| O | 14.06680000 | 1.55420000 | 4.73940000 |
| O | 14.07120000 | 11.12810000 | 8.42560000 |
| O | 0.79260000 | 14.85790000 | 7.33690000 |
| O | 14.07120000 | 1.81300000 | 7.33690000 |
| Fe | 5.77210000 | 14.73490000 | 5.91230000 |
| O | 5.77210000 | -0.15310000 | 6.74450000 |
| Fe | 14.07120000 | 10.00940000 | 4.83330000 |
| Fe | 0.79260000 | 14.72550000 | 2.82820000 |
| Fe | 2.31710000 | -1.27960000 | 2.26480000 |
| O | 14.07120000 | 8.12280000 | 5.08980000 |
| O | 2.45240000 | 13.61610000 | 2.32000000 |
| O | 14.07120000 | 8.64430000 | 7.67980000 |
| Fe | 4.03830000 | 14.70710000 | 2.48530000 |
| O | 5.60010000 | 0.55570000 | 2.05070000 |
| Fe | 7.43190000 | -0.03010000 | 8.16900000 |
| Fe | 8.58630000 | 14.62960000 | 6.75450000 |
| O | 7.43190000 | 14.85790000 | 7.33690000 |
| Fe | -0.86720000 | 1.69000000 | 5.91230000 |
| Fe | -0.86720000 | 14.73490000 | 5.91230000 |
| Fe | -0.86720000 | 10.01890000 | 7.91740000 |
| O | -0.86720000 | 3.57670000 | 5.65580000 |
| Fe | 7.43190000 | 14.72550000 | 2.82820000 |
| O | 9.11620000 | 0.56510000 | 2.19740000 |
| Fe | -2.52710000 | 14.72550000 | 2.82820000 |
| Fe | 10.75150000 | 14.72550000 | 2.82820000 |
| Fe | -1.18430000 | 11.67270000 | 2.31060000 |
| Fe | -0.86720000 | 7.01350000 | 4.58160000 |
| O | -0.86720000 | 10.41620000 | 6.00790000 |
| O | -0.86720000 | 0.57120000 | 2.32000000 |
| O | 12.41140000 | 0.57120000 | 2.32000000 |
| O | 12.41140000 | 13.61610000 | 2.32000000 |
| O | -0.89760000 | 10.18000000 | 3.41980000 |
| O | -0.86720000 | 6.06040000 | 6.40180000 |
| O | 12.41140000 | -0.15310000 | 6.74450000 |
| H | 5.77210000 | 3.43160000 | 3.96660000 |
| H | 7.53400000 | 2.70320000 | 8.39060000 |
| H | 12.41140000 | 3.43160000 | 3.96660000 |

1. *Corresponding author phone: (86) 10-62333305; fax: (86) 10-62333305; e-mail: wangfei@ustb.edu.cn [↑](#footnote-ref-1)
